# A Reduced Self-Positive Belief Underpins Greater Sensitivity to Negative Evaluation in Socially Anxious Individuals

**DOI:** 10.5334/cpsy.57

**Published:** 2021-04-28

**Authors:** Alexandra K. Hopkins, Ray Dolan, Katherine S. Button, Michael Moutoussis

**Affiliations:** 1 Wellcome Trust Centre for Neuroimaging, UCL, UK; 2 Max Plank – UCL Center for Computational Psychiatry and Ageing, UK; 3 Department of Psychology, University of Bath, UK

**Keywords:** computational psychiatry, associative learning, belief update, social anxiety

## Abstract

Positive self-beliefs are important for well-being, and are influenced by how others evaluate us during social interactions. Mechanistic accounts of self-beliefs have mostly relied on associative learning models. These account for choice behaviour but not for the explicit beliefs that trouble socially anxious patients. Neither do they speak to self-schemas, which underpin vulnerability according to psychological research. Here, we compared belief-based and associative computational models of social-evaluation, in individuals that varied in fear of negative evaluation (FNE), a core symptom of social anxiety. We used a novel analytic approach, ‘clinically informed model-fitting’, to determine the influence of FNE symptom scores on model parameters. We found that high-FNE participants learn more easily from negative feedback about themselves, manifesting in greater self-negative learning rates. Crucially, we provide evidence that this bias is underpinned by an overall reduced belief about self-positive attributes. The study population could be characterized equally well by belief-based or associative models, however large individual differences in model likelihood indicated that some individuals relied more on an associative (model-free), while others more on a belief-guided strategy. Our findings have therapeutic importance, as positive belief activation may be used to specifically modulate learning.

**Author Summary:**

Understanding how we form and maintain positive self-beliefs is crucial to understanding how things go awry in disorders such as social anxiety. The loss of positive self-belief in social anxiety, especially in inter-personal contexts, is thought to be related to how we integrate evaluative information that we receive from others. We frame this social information integration as a learning problem and ask how people learn whether someone approves of them or not. We thus elucidate why the decrease in positive evaluations manifests only for the self, but not for an unknown other, given the same information. We investigated the mechanics of this learning using a novel computational modelling approach, comparing models that treat the learning process as series of stimulus-response associations with models that treat learning as updating of beliefs about the self (or another). We show that both models characterise the process well and that individuals higher in symptoms of social anxiety learn more from negative information specifically about the self. Crucially, we provide evidence that this originates from a reduction in the amount of positive attributes that are activated when the individual is placed in a social evaluative context.

## Introduction

‘We don’t see things as they are, we see things as we are’ – Anaïs Nin

We tend towards optimism instead of realism, often overestimating our competence and likeability (Sharot, Korn, & Dolan, 2011). This bias appears useful, allowing individuals who hold a positive self-view to benefit from better psychological well-being and mental health (Conversano et al., 2010; Korn, Sharot, Walter, Heekeren, & Dolan, 2014; Moore & Fresco, 2012). One’s self-view is theorised to be shaped by interpersonal interactions and the perceptions we think others have of us (Beck, 1971, 2008; Cooley, 1902; Will, Rutledge, Moutoussis, & Dolan, 2017). The nature of the social information individuals receive, and what they do with that information, is key to understanding how self-beliefs develop and are maintained (Spence & Rapee, 2016).

Cognitive theories of depression and social anxiety hold that repeated exposure to social adversity can teach an individual that the world is an unpredictable and hostile place, where they should expect criticism and poor social outcomes (Beck, 2008; Clark & Wells, 1995). This negative learning forms the schema, a system of beliefs and expectations through which future self-relevant social information is processed (Clark & Wells, 1995; Rapee & Heimberg, 1997). Once activated, the self-schema acts as an information filter, influencing attention, perception, learning and memory, such that the dysfunctional self-views are maintained (Beck, 2008). Schemas are disorder-specific; for social anxiety, their content relates to the core fear of being negatively evaluated by others.

It is important to understand the psychological mechanisms behind inferring evaluation of self and others, and how this integrates into our self-schema. Evidence indicates that the activation of self-beliefs, or self-schema, and the updating of such beliefs in response to social feedback is key (Button, Browning, Munafò, & Lewis, 2012; Korn, Prehn, Park, Walter, & Heekeren, 2012; Korn et al., 2014). However, temperamental preparedness and operant learning routes to anxiety, such as behavioural inhibition and reinforcement via safety-behaviours, are also postulated to be important (Spence & Rapee, 2016).

Individuals who show high fear of negative evaluation (FNE) display negatively biased processing of social-evaluative information (Winton, Clark, & Edelmann, 1995) and are prone to social anxiety (Stopa & Clark, 2001). Button and colleagues (Button et al., 2012, 2015) demonstrated negative bias about the self in a Social Evaluation Learning Task, wherein a computer persona described either themselves or an unknown other. Those more fearful of negative evaluation selected significantly fewer positive attributes when asked to predict how the computer persona would describe them, but displayed no bias when making predictions about unknown others. The fact that this negative bias specifically manifested when evaluations are related to the self, suggests that individuals integrate social information differently depending on the context and focus of the evaluation, which is consistent with the cognitive models (Beck, 1971; Cooley, 1902).

Computational cognitive studies have recently addressed self-evaluation (Koban et al., 2017; Will et al., 2017). So far, studies have mostly relied on associative learning models (Rescorla & Wagner, Allan, R, 1972) to capture phenomena such as healthy people giving more weight to positive, rather than negative, information about themselves. Koban et al. (2017) analysed self-evaluation using an associative model, to test whether learning rates – *association values* in learning theory (Hill, 1960) – depended on social anxiety. Social Anxiety Disorder patients were found to have higher learning rates for negative attributes about themselves, compared to healthy controls. Learning-rate based models give a good description of changes in moment-to-moment evaluation of the self, but learning rates are not stable psychological characteristics, depending on a host of factors (Browning, Behrens, Jocham, O’Reilly, & Bishop, 2015; Dorfman, Bhui, Hughes, & Gershman, 2019; Mathys, Daunizeau, Friston, & Stephan, 2011). Clinically, this maleability is useful, opening up maladaptive learning rates to therapeutic intervention (Kube et al., 2019).

Instead of focusing on behaviour assumed to be gradually reinforced, belief-based frameworks focus how evidence, here provided by social information, updates beliefs. This framework can accommodate the top-down role of self-schema/beliefs, including trait-like views about the self activated given a social context, more naturally than associationist approaches. It also explicitly accounts for the role of uncertainty, which may be especially important for social learning (Kruschke, 2008).

A Bayesian approach is particularly well suited to modelling the top-down influence of beliefs (Stankevicius, Huys, Kalra, & Seriès, 2014), as it has belief update at its core and explicitly represents different strengths of belief. For example, I may believe that I am ‘80–90%’ socially competent but also allow for a socially incompetent 10–20%. Alternative beliefs are then strengthened or weakened as social information accumulates. The certainty of beliefs is informed by learning throughout an individual’s history. Certainty then determines how open existing (‘prior’) beliefs are to change, i.e. determines learning rates. Intuitively, someone with a negative self-view may be more likely to integrate negative evaluations, as they are more in line with their own initial beliefs (see SI for a tutorial demonstration). Similarly biased belief-updating has been demonstrated in non-social reward-based tasks (Stankevicius et al., 2014).

We aimed to clarify the explanatory power of these two psychological frameworks in social-evaluation. We expected associative learning models to capture well the dynamics of learning, while a Bayesian cognitivist framework would provide insight into how beliefs evolve and affect learning. We were interested in mechanisms of biased learning in individuals with high fear of negative evaluation, and its potential basis in biased updating of beliefs about the self.

## Methods and Materials

### Measures

**Figure 1:**
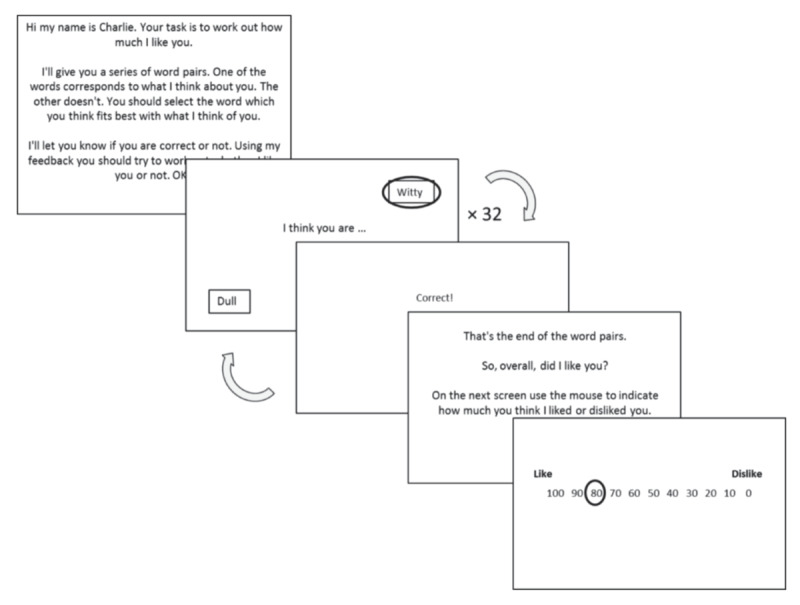
Each task block consisted of 32 trials. Participants had to choose between positive and negative words. There were 6 blocks in total, corresponding to 6 evaluative conditions, termed *personas* – Self-like, self-neutral, self-dislike, other-like, other-neutral, other-dislike. Self/other refers to who is being evaluated, like/neutral/dislike refers to the probability of a positive word being correct (0.8, 0.5, 0.2 for the like/neutral/dislike rules respectively).

Published data was obtained from (Button et al., 2015). Data consisted of a Social Evaluation Learning Task (***Figure 1***) completed by 100 participants and a range of questionnaires, of which the primary measure was the Brief Fear of Negative Evaluation (BFNE) scale (Leary, 1983). A higher BFNE score indicates greater fear of negative evaluation. For a full details of the task and sample please see (Button et al., 2015).

### Sample

In line with a dimensional approach to psychopathology, the original study recruited participants with a range of social anxiety symptoms using an efficient sampling approach to over recruit from the maximally informative extremes (high or low symptoms), ensuring a third of participants had scores in the bottom quartile of BFNE scores, a third from the top quartile and a third from the mid-range using random sampling to exclude one out of two participants with mid-range scores. Participants completed the diagnostic CIS-R (Lewis, Pelosi, Araya, & Dunn, 1992), which provides diagnoses in line with ICD-10 and DSM-IV. Seven participants met the diagnostic criteria for social phobia and 62 exceeded the cut-off for clinically significant social anxiety on the BFNE.

### Associative and belief-based models

To assess how choices evolved as a function of social feedback, we used computational models. We formalised how social feedback influenced subsequent choices about the self and other using adapted Rescorla-Wagner reinforcement learning models (Rescorla &Wagner, Allan, R, 1972) and novel belief-update models. Here we describe the key features of the models, with technical details to be found in the Supplement.

#### Associative Learning models

Associative learning models describe learning in terms of value. Here, participants learn the value of the action ‘choose the positive attribute’ or ‘choose the negative attribute’, based on feedback. These action-values *Q*(*action,context*) are updated after each outcome. A discrepancy between choice and outcome forms a ‘prediction error’, *PE*. The *PE* is then multiplied by a learning rate, *λ_c_*, a parameter weighing the impact of new evidence on existing values, and the result added to update the existing action-value. High learning rates correspond to new evidence having a strong impact, quickly replacing old learning. The *context s_t_* simply indexes which state, i.e. computer persona *×* (self vs. other), the trial *t* was about.


1 \[
\begin{array}{*{20}{c}}
{P{E_t} = {r_t} - {Q_{t - 1}}({a_t},{s_t})}\\
{{Q_t}({a_t},{s_t}) = {Q_{t - 1}}({a_t},{s_t}) + {\lambda _c}\;P{E_t}}
\end{array}{\mathrm{ }}
\]

We focused on learning rates, as these easily characterise which conditions have a major or minor impact on learning. Following Koban et al. (2017), we expected that learning could be valence dependent and therefore allowed separate learning rates for trials with a positive or negative outcome word (irrespective of what choice led to it). So, people might have *λ_+ve outcome_* > *λ_–ve outcome_*. Based on the descriptive findings of Button et al. (2015), we were interested in self/other distinction and therefore considered models that had separate learning rates depending on whether the object of learning was self or other, giving *λ_self,+ve_, λ_other,+ve_, λ_self,–ve_* etc. Models could include an initial value parameter, allowing starting values *Q*(*+ve word,s_t=_*_0_) to reflected an individuals starting tendency towards positivity.

Actions were chosen probabilistically, as a function of a propensity variable for choosing each action. This propensity was the action value *Q*(*a,s*) biased by a ‘positivity bias’ ρ, which quantified biases in favour of choosing positive attributes independent of learning (Eq. 2). *Q(a,s)+*ρ then entered a standard softmax function, weighed by a ‘decision noise’ parameter τ > 0:


2 \[
\begin{array}{*{20}{c}}
{P(a = + veword;s) = z\;exp\frac{{Q(a,s) + \rho }}{\tau }}\\
{P(a = - veword;s) = z\;exp\frac{{Q(a,s)}}{\tau }}
\end{array}{\mathrm{ }}
\]

Where *z* ensured that probabilities add up to 1.

#### Belief-update models

Belief-update models conceptualised participants as holding beliefs about how approving each computer persona was, from 0 to 1. Such beliefs do not contain just one value (‘this persona will give me 80% +ve attributions’) but also embody an uncertainty (‘but it could be 70 to 90%). They can be formalized by a beta distribution, which conveniently describes beliefs through the amount of positive evidence *α* and that of negative evidence β held in mind. The mean probability of approval is then the average *p* = α/(α + β).

The belief parameters were updated in every round by augmenting the evidence corresponding to the outcome (say, positive) by 1 piece of evidence. However, we sought to also model views about the self that participants brought to bear independent of learning. Greatly simplifying clinical theory (Pinto-Gouveia, Castilho, Galhardo, & Cunha, 2006), we represented this as the positive and negative evidence people brought to bear. People thus held two belief components. The first was trait-like, (α*_trait_*, β*_trait_*), parameterized individual variability. It was fixed for the duration of the task, and represented the self- or other- view activated given the current context^1^. The second was state-like, (α*_state_*, β*_state_*), and it accumulated task information.


3 \[
\begin{array}{*{20}{c}}
{{\alpha _t} = {\alpha _{trait}} + {\alpha _{state,t}}}\\
{{\beta _t} = {\beta _{trait}} + {\beta _{state,t}}}
\end{array}{\mathrm{ }}
\]

Next, we considered that individuals may not integrate an indefinite amount of evidence, instead gradually discarding older task information. Memory decay parameters 0 *<* η *<* 1 thus quantified a participant’s effective working memory. Belief-update models could include separate initial values α*_state, t_*_=0_, β*_state, t=_*_0_. They could also be separated into self/other with respect to α*_trait, self_*, α*_trait, other_* etc., and with respect to initial values, or indeed the memory decay parameter.

Belief distributions inherently contain uncertainty, which can affect decision variability (Moutoussis, Dolan, & Dayan, 2016). Hence, we considered two classes of probabilistic action choice. In the first, point estimates such as the mean of a belief distribution was used to determine policy. Here, choice variability was independent of belief uncertainty. In the second class, reduced belief uncertainty as a result of evidence accumulation resulted in reduced decision variability. We thus considered several ‘link functions’ from belief to choice (see Supplement), and determined the best by model comparison. The winning action-choice function was the one which only depended on the mean of the belief distributions (Eq. 4):


4 \[
P(a = + veword;context = s) = z\;exp\frac{{{\alpha _s}}}{{({\alpha _s} + {\beta _s})\tau }}
\]

A short summary of all models is displayed in ***Table 1***. Detailed descriptions are given in the Supplement.

**Table 1:** Model families, grouped according to their defining core parameters.


MODEL FAMILY NAME	NP	CORE PARAMETERS	ADDITIONAL PARAMETERS	

Valence model – 2λ	3–5	*λ_+ve_, λ_–ve_*, τ	Initial bias	Pos. bias

Self/other asymmetric valence – 3λ	4–6	*λ_self pos_, λ_self,–ve_, λ_other_*, τ	Initial bias	Pos. bias

Self/other valence – 4λ	5–7	*λ_self,+ve_, λ_self,–ve_, λ_other,+ve_, λ_other,–ve_*,τ	Initial bias	Pos. bias

Belief-update	4	α, β, η, τ		

Belief-update self/other	7	*α_self_, β_self_, α_other_, β_other_, η_self_, η_other_*, τ		

Belief-update self/other initial bias	9	*α_self_, β_self_, α_other_, β_other_, η_self_, η_other_*, τ	α*_initial_*	β*_initial_*


*Note*: The ‘Additional parameters’ were used to optimize fit within each family and hence estimation accuracy for the parameters of core interest. NP gives the range number of parameters in each family, i.e. with or without parameters described as ‘additional’.

#### Modelling the relation to Fear of Negative Evaluation

We fitted all models using a hierarchical procedure that optimizes estimation of the relation between model parameters and symptomatic measures, i.e. by *clinically informed model-fitting*. Traditional hierarchical modelling reduces noise in parameter estimates, but we have found that empirical (population) priors which do not take adequately into account the possible correlations with external measures can increase the rates of Type 1 or Type 2 error, in subsequent correlation analyses with unmodelled psychometric measures (Moutoussis, Hopkins, & Dolan, 2018). Here, incorporating key psychological hypotheses in the model-fitting can give more accurate estimates of the relationship between model parameters and BFNE scores. As in traditional hierarchical modelling, individual parameters were estimated by taking into account the population distribution they came from, i.e. the ‘group prior distribution’. This was in turn estimated from the data, including BFNE scores. We embedded FNE into model-fitting by including slope parameters that estimated a linear contribution of BFNE scores on the mean of the population distribution whence individuals were sampled from, as detailed below.

Let θ be a cognitive parameter that may correlate with BFNE. We modelled this correlation as a linear relationship between BFNE and the mean of θ over people with that value of BFNE:


5 \[
\begin{array}{*{20}{c}}
{\theta \tilde\ N({\mu _\theta }(FNE),\sigma )}\\
{{\mu _\theta }(FNE) = w\;BFNE + {\theta _0}}
\end{array}{\mathrm{ }}
\]

Where *θ*_0_ is an intercept and in the first instance *σ* is taken to be independent of FNE. As a cognitive model is fitted using Eq. 5, the posterior distribution over the slope parameter *w* can be estimated, providing the credible interval over the dependence of of *θ* on FNE.

We fitted the learning models under consideration (***Table 1***) using RStan (Carpenter et al., 2017). Following RStan convention, means over population-level parameters were scaled so as to be sampled from a standard normal distributions. The respective standard deviations were sampled from half-Cauchy distributions. The individual-level parameters were appropriately constrained in their native space (e.g. 0–1 for learning rates), then transformed so as to be subject to the Gaussian distributions informed by the relevant group priors. We initialised Markov-Chain Monte Carlo chains with random starting values. Posterior distributions were formed after 1000 burn-in samples from 4 chains, resulting in a total sample size of approximately 8,000. Convergence was determined by visual inspection of the trace plots and monitoring the Gelman-Rubin statistic for each parameter (Gelman & Rubin, 1992), with values close to 1.00 implying convergence.

We compared the goodness of fit of different models via approximate leave-one-out cross-validation (Loo). This provides a measure of the likelihood of left-out data, suitable for estimating model-fit in hierarchical models (Carpenter et al., 2017). We then examine the credible intervals of correlation parameters (*w* above) between BFNE and specifically hypothesized parameters (learning rates, beliefs about the self and others) separately in the winning associative and belief-based models. A hypothesis that a parameter correlated with BFNE was tested by determining whether the credible interval of *w* included zero.

## Results

### Model fitting and model comparison

Model comparisons using left-out likelihood (LOO) (Vehtari, Gelman, & Gabry, 2017) showed that associative learning models that included separate learning rates for self outperformed ones that did not distinguish between agents. There were also big improvements in model fit upon including an initial bias parameter that allowed individuals to vary in an initial propensity to choose a positive word, and upon including a constant ‘positivity bias’ boosting the action-value of positive information. Although the best-fitting associative learning model in absolute terms was the self/other valence model, LOO model comparison indicated weak evidence for this model over the next best-fitting model with fewer parameters. We thus also took account parameter recoverability, which was enhanced by having fewer parameters. We thus selected for further work the ‘self/other asymmetric valence model’, with 3 learning rates, an initial bias parameter and a positive bias parameter (see Supplementary Information for details of the full self/other valence model).

As shown in ***Table 2***, the best-fitting model overall was a belief-update model with separate self/other alpha, beta and memory parameters and also had free initial bias parameters which also included starting beliefs to vary between individuals. Again, LOO model comparison indicated weak evidence for this model. Following a similar rationale as for the associative models, we selected for further work a ‘separate self/other’ model with a shared memory parameter. The belief-update model without separate initial *α* and β parameters also performed almost as well as the best models in their respective families. However, the parameters involved might relate to our hypotheses regarding self-Other activated schemata, and hence we proceeded simply with the best-LOO models. Belief models with separate ‘trait’ parameters for self and other performed much better than models without, emphasizing a necessary distinction between self and other in learning. We include more details for all models considered above in the supplement.

**Table 2:** The best models from each family according to approximate leave-one-out cross-validation. Final models selected are given in bold.


MODEL FAMILY NAME	N. PARAM	LOO

Valence	3–5	–10026

Self/other valence	5–7	–9858

**Self/other asymmetric valence**	**4–6**	**–9862**

General learning rate	3–5	–9966

Belief-update IB	7	–9954

**Belief-update self/other IB**	**8**	**–9768**

Belief-update self/other full IB	9	–9762


*Note*: IB refers to models with Initial Bias parameters.

**Figure 2:**
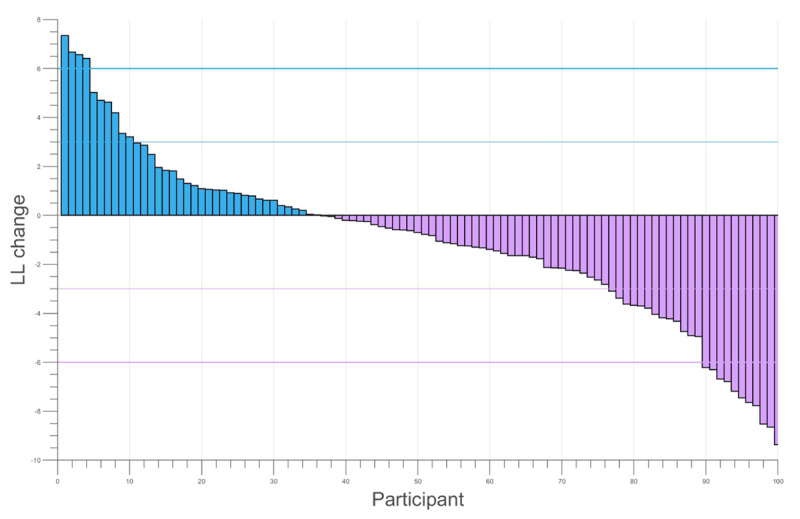
Individual log likelihoods for associative learning vs belief-update model. Positive values indicates greater evidence for the associative learning model. The horizontal bars indicate log likelihood differences of +/–3 and +/–6, conventionally mild and strong evidence in favour of one model over the other.

Although the belief-based model had better fit statistics overall, we asked whether this was because it fitted most people better than the associative models, or whether those that were better described by associative models were in the minority. To estimate this, we simply examined the distribution of the difference between maximum-likelihood (ML) estimates for the associative vs. belief-based models, shown in ***Figure 2***. This indicates that for the majority of participants there was no clear difference between the models, but for about a fifth there was conventionally strong evidence that one or the other model gave a better account of the data. We did not find a significant correlation between BFNE score and the belief-associative ML difference. Here, we computed the difference in log-likelihoods between the two models, with larger differences indicative of one model describing the data better than the other. There was no significant correlation of log-likelihood with BFNE score when models were analysed separately either.

### The relationship between BFNE and model parameters

Based on the literature (Carpenter et al., 2017) and the theory of self-schema, we examined the specific hypotheses that BFNE would relate to the trait-evidence in the self schema (*α_self_* and/or *β_self_*) or the corresponding learning rates *λ_self,+ve_* and *λ_self,-ve_* (See supplement for the theoretical derivation of this approximate correspondence). We also examined in an exploratory manner whether the other parameters of the winning models correlated with BFNE scores. We assessed each of the BFNE weight parameters to determine whether their credible interval overlapped 0, which would not support an effect of BFNE on that parameter (***Table 3***).

**Table 3:** Parameter weights on FNE, derived from clinically informed model-fitting.


Associative Learning parameter	Mean *w* [Lower CI – Upper CI 95%]	Belief-update parameter	Mean *w*[Lower CI – Upper CI 95%]

*λ_self,+ve_*	0.01 [–0.09 0.09]	*α_self_*	**–0.47 [–0.87 –0.06]**

*λ_self,–ve_*	**0.11 [0.02 0.20]**	*β_self_*	–0.24 [–1.55 1.08]

*λ_other_*	–0.05 [–0.19 0.09]	*α_other_*	–0.02 [–0.16 0.19]

τ	–0.07 [–0.01 0.15]	*β_other_*	0.07 [–0.31 0.45]

Initial bias	–0.09 [–0.19 0.01]	η	–0.22 [–0.56 0.13]

Pos. bias	–0.09 [–0.19 0.01]	τ	–0.09 [–0.25 0.06]

		*α_initial_*	–0.39 [–0.99 0.22]

		*β_initial_*	–0.97 [–5.07 3.13]


^a^ *Note*: Mean weights and 95% credible intervals for self/other valence model and self/other belief-update model are shown, with intervals not containing zero shown in bold.

The only associative weight parameter that did not have credible intervals including zero was for the self-negative learning rate (see ***Table 3***). This weight parameter was positive, indicating the higher the individual is in FNE, the larger the self-negative learning rate will be. Therefore, it appears that in an associative learning framework, fear of negative evaluation is specifically related to over weighting of negative information, while positive information processing appears intact.

The only belief-update weight parameter that did not have credible intervals including zero was was between BFNE score and the *α_self,+ve_* parameter (see 3). This weight parameter was negative, indicating the higher the individual is in FNE, the lower the amount of positive evidence in the self-schema, *α_trait,self_*, will be. The more negative balance of the self-schema then decreases the mean belief in approval in individuals with higher FNE.

We then explored whether the best fitted parameter values provided evidence for the theoretical correspondence between the two models. From the MLE fit parameters, indeed, *α_trait,self_* was strongly anticorrelated with the *λ_–ve,self_*, Spearman *r* = –0.49, raw *p =* 3.006*e–*07 and *β_trait,self_*, Spearman *r = –*0.3, raw *p <* .01 (Spearman’s rho was used due to non-normality). *λ_–ve,self_* was also correlated with *β_trait,other_*, Spearman *r = –*0.21, *p =* 0.04, but none of the other parameters of the belief-model. Finally, *λ_–ve,self_* was also strongly anticorrelated to the *proportion* of activated positive self-beliefs, represented by the mean of the beta distribution (Spearman *r = –*0.27*, p <* 0.01), although this is of not, of course, an independent relationship. The best fitted parameter values from the MCMC fits indicated an even stronger relationship, with the key parameters *α_trait,self_* being strongly anticorrelated with the *λ_–ve,self_*, Spearman *r =* –0.85, raw *p =* 1.5349*e–*29, giving evidence that people with larger learning rates for self-negative information also have lower positive self-belief. Again, there was a strong relationship between the *λ_–ve,self_* parameter and the proportion of activated positive self-beliefs derived from the mean of the self beta distribution, Spearman *r = –*0.78, raw *p =* 4.4583*e–*22. There was also a positive correlation between the initial bias and *α_trait,self_* parameter, suggesting they represent similar concepts (Spearman *r =* 0.50, *p <* .001) and suggesting people with lower positive self-belief have a prepotent starting tendency towards more negative responses. None of the other parameters indicated correlations.

### Generative Performance

Crucially, good models not only statistically fit the data overall, but are also able to capture specific data features of interest that have not been privileged during modelling (Palminteri, Wyart, & Koechlin, 2017). We therefore tested this using our best-fit models. The best associative learning model and belief-update models were used to generate pseudo-data from 100 sample datasets consisting of 1000 participants each, simulating ‘ideal experiment’ conditions, here with more subjects than resource constraints allow. We checked whether these synthetic experiments reproduced the published findings from real people Button et al. (2015), ran the same formal statistical tests, and examined the credible intervals of each result over simulated samples. We computed the percentage positive response for each persona from the generated data as the number of positive word choices made/32 (number of trials). We ran linear mixed effects (LME) analyses including BFNE scores, persona (like/neutral/dislike) and referential condition (self/other) as predictor variables and percent positive response as outcome variable.

**Figure 3:**
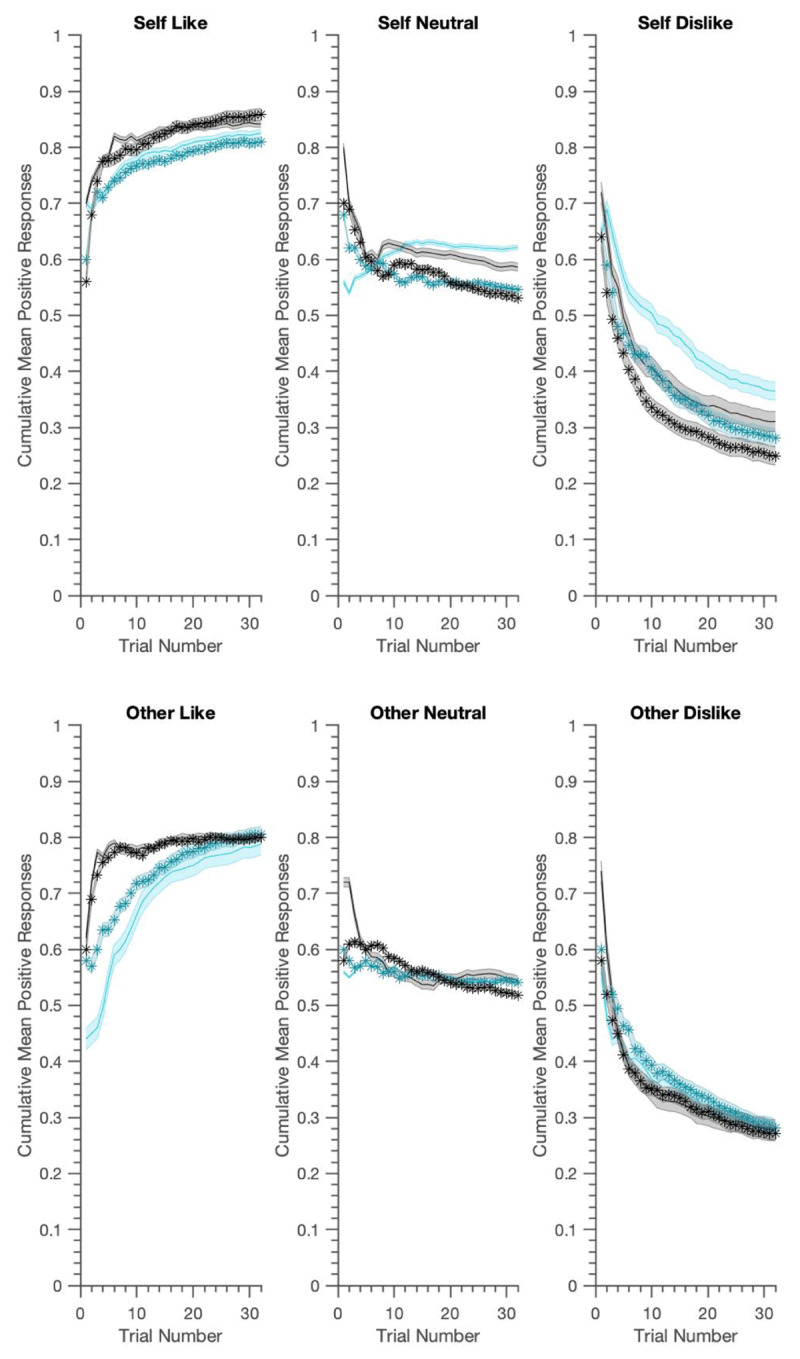
Generative performance for the Associative Learning S/O asymmetric model; mean cumulative positive words chosen for actual data (in black) vs. data generated from ‘clinically informed fitting’ (cyan). Data is visualised using median-split FNE scores (lighter=lower BFNE) and shaded zones represent +/– SEM. The generated data captures the asymmetries in positive vs. negative word selection and the group differences between high and low FNE for the self-referential condition well. There is slower initial learning, especially in the like condition and this model chooses over-optimistically, especially in ‘dislike’ conditions.

**Figure 4:**
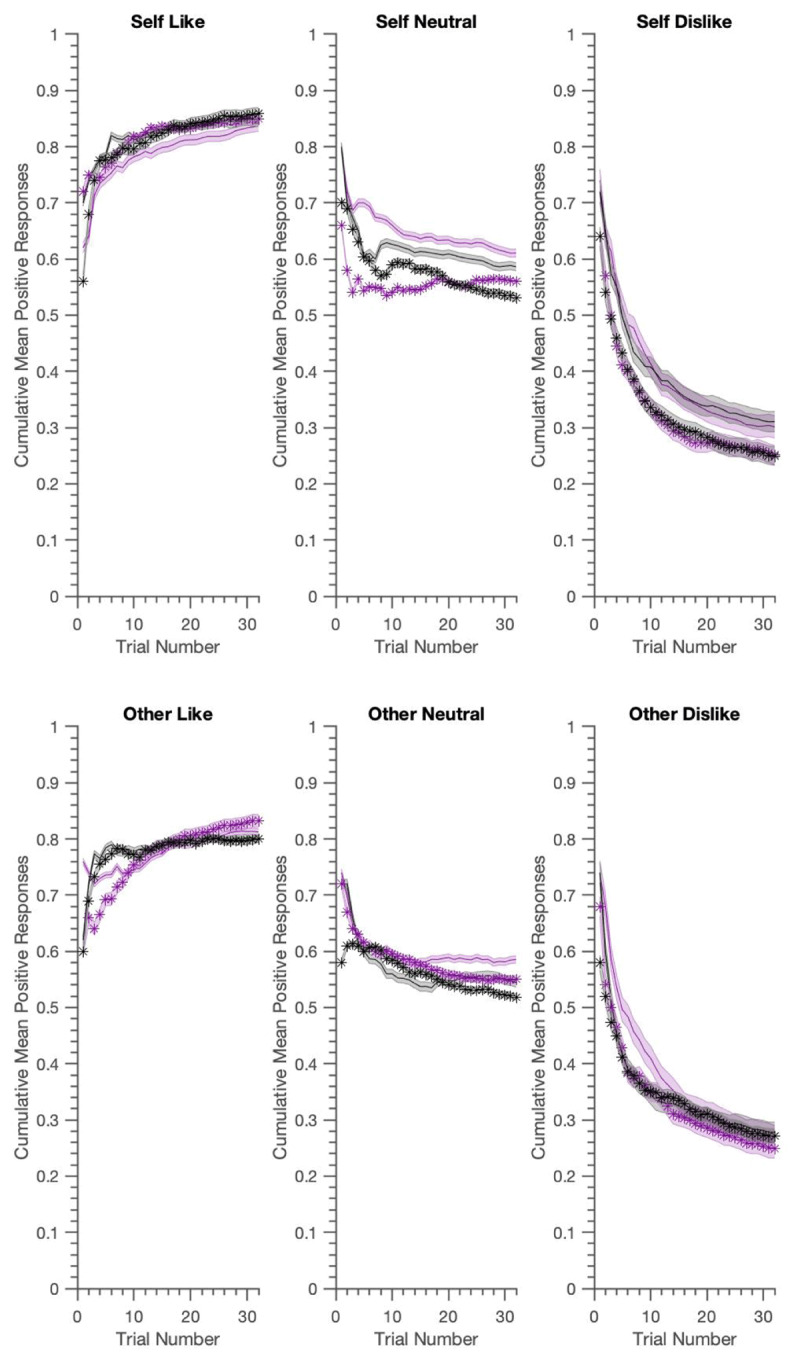
Generative performance for the Self/Other Belief-Update model; mean cumulative positive words chosen for actual data (in grey) vs. model (mauve). Again data is visualised using median-split FNE scores, with shaded zones representing +/– SEM for high (darker shade) vs. low (lighter shade) BFNE scores. The generated data captures well the asymmetries in positive vs. negative word selection and the group differences between high and low FNE for the crucial self-referential dislike condition.

As illustrated in ***Figures 3*** and ***4***, the generated data reproduced most key features of the real experiment. ***Table 4*** shows that the LME results presented in (Button et al., 2015) were well reproduced. Using generated data from the belief-update model, we replicated almost all of the main and interaction effects in over 95% of the samples. The three-way interaction, however was slightly underestimated. The associative learning model did better in this regard, not only replicating all of the main and interaction effects, but also providing evidence for the significant three-way BFNE *×* persona *×* condition interaction in over 95% of the samples. Both models slightly overestimated the BFNE difference for the neutral condition.

**Table 4:** Generative performance statistics.


CONTRAST	ASSOCIATIVE LEARNING MODEL MEAN *β* COEFFICIENT	% OF SIG SAMPLES	BELIEF-UPDATE MODEL MEAN *β* COEFFICIENT	% OF SIG SAMPLES

Main effect BFNE	–0.74 [–0.75 –0.73]	100	–0.73 [–0.74 –0.72]	100

Main effect self/other	–13.28 [–13.56 –13.00]	100	–13.52 [–13.84 –13.20]	100

Main effect persona: like	21.55 [20.98 22.11]	100	24.20 [23.53 24.88]	100

Main effect persona: neutral	19.36 [18.57 20.16]	100	15.97 [15.22 16.73]	94

BFNE × self/other	0.32 [0.32 0.33]	100	0.28 [0.27 0.29]	100

BFNE × persona: like	0.74 [0.73 0.76]	100	0.70 [0.68 0.71]	100

BFNE × persona: neutral	0.19 [0.17 0.20]	34	0.26 [0.25 0.28]	61

BFNE × self/other × persona	–0.30 [–0.31 –0.29]	100	–0.23 [–0.24 –0.21]	89


^a^ *Note*: [Lower CI Upper CI 95%].

## Discussion

We aimed to understand learning about self and others in those fearful of social evaluation, by formalizing and comparing two classic psychological perspectives. This is important, as the way in which belief-based accounts used by clinicians should be formalized is unknown, as is how valid they are and whether they are distinct from associationist accounts. Using a well-established Social Evaluation Learning task, we provide evidence that reduced positive content within activated self-schemata underpins increased sensitivity to negative evaluation in socially anxious individuals. Individuals with a less positive self-schema also had a larger self-negative learning rate when investigated using the associative framework. Both associative learning and belief-based models described social learning well, with belief-models especially able to capture the interaction between task context and participant disposition.

We replicated, and also refined, influential findings on associative learning in social anxiety (Koban et al., 2017). Using a task with evidence for reproducibility at the psychological level (Button et al., 2012, 2015), we reproduced the model results reported in (Koban et al., 2017). Namely, socially anxious (high-FNE) individuals had higher learning rates governing the impact of negative information on predictions about the self. We finessed this associative account by including a ‘positivity parameter’, thus better accounting for participants’ optimism bias (Sharot et al., 2011). We also showed that learning rates for positive and negative feedback for the other-referential context were not distinguishable from each other, further pointing at the relevance of self-bias in social anxiety.

Detecting the dependence of task parameters on FNE in this subclinical sample was established through *clinically informed model-fitting*, which makes use of a fundamental property of hierarchical statistical models. These infer the characteristics of each individual not only from the data they provided, but also from the specific population from which they are drawn. Clinically informed model-fitting allowed (yet did not force) empirical priors over cognitive parameters like learning rates to be informed by clinical data, here BFNE scores (Moutoussis et al., 2018). It thus allowed more accurate estimation of the correlation between parameters and FNE. Research is starting to benefit from clinically informed model-fitting (Brown, Chen, Gillan, & Price, 2020).

To examine whether key features of successful associative learning models were understandable in terms of self-beliefs, which statistically account for improvement during therapy (Gregory & Peters, 2017), we formulated a very simple model of social belief update. We assumed that upon entering a context of evaluation of self or other, individuals activate beliefs about themselves (or others), over and above the evidence gleaned during the task. We focues on the trait-like component of activated schemata, which are constant for the duration of the task but may differ according to the contextual focus of evaluation. This activated self-schema consisted of positive and negative ‘notional’ evidence that each individual brought to mind. We hypothesised that ordinary beliefs could be modelled as Bayesian beliefs, so that the strength of belief could be quantified much like in CBT (‘I believe 70–80% that I will be judged positively’). This meant that belief change not only depended on evidence, but also on the certainty of prior beliefs (Moutoussis et al., 2016). Overall, the success of belief-based models suggested that this was indeed the case. Next, we hypothesized that the amount of evidence that each individual processed would be variable, in effect a working memory capacity. Again, the evidence supported this hypothesis. Another ‘signature’ of belief-based cognition might be that more uncertain participants would show increased decision variability. However, model comparison provided evidence against this.

Most importantly, FNE was predicted by the amount of positive evidence about the self that was held in mind independent of task feedback. The variation in this positive self-evidence accounted for almost half the variance in self-negative learning rates. This was not, however, the only important model feature, as there was also evidence for reduced negative self-evidence. Combined, these two features may mean that social anxiety is associated with greater uncertainty in one’s beliefs about the self. Such increased uncertainty would predict lesser stability of self-evaluation, reminiscent of the changeable self-evaluation found in individuals with low self-esteem (Will et al., 2017). Importantly, the proportion of positive to negative self-evidence was greater in those with lower self-negative learning rates. Thus an activated self-schema including more positive evidence correlated strongly with diminished association value for negative attributes, largely reconciling cognitivist and behaviourist perspectives.

Leave-one-out cross-validation measures suggested that belief-based models may give a better account of behaviour overall, but this finding is likely to hide important individual differences in learning mechanisms. Preliminary analyses indicated that a minority of individuals substantially favoured associative learning, while others belief-updating. Belief-based models are a simple case of model-based cognition, updating the probability of a transition in the environment (that a persona will judge one positively), while the association models are model-free, incrementally associating values to actions. Thus, some people may be more model-based, whereas others more model-free in the domain of self-evaluation, as people are in impersonal cognition (Daw, Gershman, Seymour, Dayan, & Dolan, 2011; Shahar et al., 2019).

Our study has the potential to inform treatments for social anxiety. Simple tasks, like the one used here, may assess both the extent of biases and also the patient’s predominant cognitive style (belief-based or associationist). Importantly, we describe cognitive mechanisms quantifying and lending support to self-schema theories of social anxiety, reproducing several features of self- and other- evaluation between groups with high and low fear of negative evaluation. Clinically, our results point towards strengthening psycho-education by incorporating rigorous research showing that patients are excessively influenced by negative feedback. In therapy, patients may benefit by learning to activate positive evidence about themselves ‘on line’, specifically upon exposure to negative feedback, consistent with the work of Kube et al. (2019). Ideally, however, testing such interventions should be guided by a reliable estimate of each individual’s cognitive parameters, rather than by features of their condition in general. Here, as is often still the case with computational analyses, further progress is needed (Enkavi et al., 2019). Being able to quantify individuals’ self-views may also prove to be useful for assessing the deeper changes that therapy has achieved, rather than just symptomatic change (Taylor & Montgomery, 2007).

There are important limitations to the modelling employed in this study. Our models include a number of hypothesis-driven additional parameters, which aim to capture well-known psychological phenomenon, such as the optimism bias Sharot et al. (2011) or initial starting propensity towards positive or negative responses (Lockwood et al., 2018). When performing simulations to assess parameter recoverability, some parameters relevant to our hypotheses were difficult to recover. Limited recovery of the ‘initial bias parameters’ from the belief-update model and ‘positivity bias’ from the associative learning model (see supplement) suggest that our study may have lacked power to detect differences with respect to FNE with respect to these parameters. Aside from reduced power, the poor recoverability of some parameters renders the model less reliable at the individual level. Nevertheless, fit measures and synthetic data studies indicated that the more complex models, though over-parameterized given our concise data at the individual level, were best in describing the subtle differences in learning associated with FNE in our population. Future studies will need data capable of more fully constraining model parameters, and possibly alternative parameterizations of key models.

Despite the decreased reliability of specific parameters and possibly because of the increased accuracy of complex models, we are able to detect our main effects of interest, and found good recoverability for the positive self-belief and self-negative learning rate. Future studies using clinical populations with larger differences at the behavioural level could observe even greater effect sizes. Thus, our study is well able to detect group level differences in learning between the high vs low FNE groups (the main objective of the study), but poor at capturing individual level differences reliably (Shahar et al., 2019). An important consideration for our more complex models was the ability to reproduce key behavioural statistics of the data, which (Palminteri et al., 2017) recommend as a method of model falsification. Simpler models, despite showing good fit statistics, were unable to capture the key FNE group differences between self and other conditions (see supplement), thus we preferred models with good fit statistics as well as generative performance. Finally, our modeling of evidence about the self was rudimentary compared to the sophistication of clinical research on self representations(Calvete, Orue, & Hankin, 2013; Pinto-Gouveia et al., 2006). Future studies modelling self-representations could combine our hierarchical clinically informed model fitting approach with this previous work.

In conclusion, individuals who are high in fear of negative evaluation (yet not care-seeking patients) are more affected by negative social feedback, compared to those unafraid of such feedback. The robustness of typical individuals is consistent with activation of more positive beliefs about themselves independently of feedback, acting as a ‘buffer’ against developing negative expectations. If replicated, this finding can inform therapeutic interventions aiming at activating positive views of self when people are in the crucible of social judgment.

## Additional File

The additional file for this article can be found as follows:

10.5334/cpsy.57.s1 Supplementary Information. Beliefs & Associations in Social Learning.

## Footnotes

Strictly, the models only contain *notional* or effective evidence, i.e. a numerical representation of the weight of affective memories, images etc. activated in real people
