# Supplementary material for: A Reduced Self-Positive Belief Underpins Greater Sensitivity to Negative Evaluation in Socially Anxious Individuals
Source: Comput Psychiatr. 2021 Apr 28;5(1):21–37. doi: 10.5334/cpsy.57 (PMC7611100; doi:10.5334/cpsy.57)
Supplement: Supplementary Information. — Beliefs & Associations in Social Learning. [file cpsy-5-1-57-s1.pdf]

Supplementary Information

Alexandra K Hopkins[1,2], Ray Dolan, Kate S Button[3,*] & Michael Moutoussis[1,2,*]

[1] Wellcome Trust Centre for Neuroimaging, UCL

[2] Max Plank - UCL Center for Computational Psychiatry and Ageing

[3] Department of Psychology University of Bath

∗ Joint supervising authors - please see CRediT statement

Supplementary Information

Associative Learning Model Descriptions

Each associative model encoded:

- the action, the valence of the attribute that the computer persona will choose in a particular trial, $a \in \{+ve\ word, -ve\ word\}$,
- the state or context $s \in \{persona:\ like, neutral, dislike\} \times \{self, other\}$,
- and the reinforcement value of the outcome, $r \in \{+1 = correct, -1 = incorrect\}$ on each trial t.

The Valence model contained separate learning rates λ for positively and negatively valenced outcome words, a.k.a +ve and -ve information, regardless of which action led to them (and so regardless of r_t). The Valence model made no distinction between self and other.

$$Q_t(a_t, s_t) = Q_{t-1}(a_t, s_t) + \lambda_{pos}\ (r_t - Q_{t-1}(a_t, s_t)) \quad \text{for} \quad +ve\ word\ outcome \qquad (1)$$

$$Q_t(a_t, s_t) = Q_{t-1}(a_t, s_t) + \lambda_{neg}\ (r_t - Q_{t-1}(a_t, s_t)) \quad \text{for} \quad -ve\ word\ outcome \qquad (2)$$

Learning rates λ further varied as follows, and are fully listed in Table S1.

Self/other valence model: separate λs for positive and negative information, and for self and other, resulting in 4 learning rates.

Self/other asymmetric valence model: separate λs for positive and negative information for self, but only one for other (i.e. 3 learning rates).

Self-valence model: separate λ for positive information for self only, and a general learning rate for all other information, resulting in 2 learning rates.

Initial values

1. Fixed uncertainty. Models without an initial bias parameter were initialised using two different methods. Fixed uncertainty operationalised the first trial

for each persona, $q_{t=0}$ to be zero, expressing equal weight between the positive and negative word and capturing a state of equal uncertainty.

2. Initial bias free parameter. The initial bias parameter, q_0, allowed the starting expectations to vary between -1 and 1. This is applied to both self and other or to the self only and impacts the first trial for every persona.

The positivity bias, ρ, is applied to each round of the expectation update as a constant term which is allowed to vary from -1 and 1. Again, this is applied to both self and other or to the self only.

Model Fit

The fit of competing models for MLE was compared using the Bayesian and Akaike information criteria (Akaike, 1998; Schwarz, 1978) (see eq. 3) at an individual level, which were then summed over participants.

$$\begin{aligned} BIC &= -2\ln p(d|\theta_{ML}) + k\ln(n) \\ AIC &= -2\ln p(d|\theta_{ML}) + 2\,k \end{aligned} \quad (3)$$

Where k is the number of free parameters, n the number of trials, d the observed data and $p(d|\theta_{ML})$ the maximum-likelihood of the parameters given the data.

Although AIC and BIC assessments of model fit are the most commonplace reported model fit statistics, these raw values are non-interpretable on their own. Factors such as number of participants and the number of trials will influence the scale of the measures, rendering comparison across studies difficult. The scores can be standardized in order to produce the pseudo-r^2 value, which reflects the variance explained by the model relative to the log likelihood from a model of pure chance (Camerer & Ho, 1999; Daw, O'Doherty, Dayan, Seymour, & Dolan, 2006).

$$\begin{aligned} C &= log(0.5)t \\ pseudo - r^2 &= 1 - L/C \end{aligned} \quad (4)$$

We can compute the log likelihood of the chance model just by inputting the total number of trials in our experiment, $t = 192$. We can then compute the pseudo-r^2 value, by taking the ratio of the two values, where L is the log likelihood of the fit model and C is the log likelihood of the chance model for each participant.

As explained in the main text, we fit all models hierarchically and report the appropriate fit statistic for hierarchical models, LOO. Table S1 gives all fit statistics for all models. We used a model comparison procedure reported in (Vehtari, Gelman, & Gabry, 2017), which compares the expected log pointwise predictive density (ELPD) for each model. We determined the size of importance of ELPD difference by taking models greater than 5 x the SE of the estimate.

Table S1

List of associative learning models and their fit indices. Each models also had one decision variability parameter, τ.

#	Model	NP	λ	q_0	ρ	LLs	AIC	BIC	Pseudo r^2	LOO
1	Valence	3	2			-10032.06	20664.13	21641.37	0.25	-10363
2	Valence	4	2	1		-9837.75	20475.51	21778.51	0.26	-10261
3	Valence	4	2	1 (self)		-9714.82	20229.64	21532.64	0.27	-10341
4	Valence	4	2		1	-9718.61	20237.22	21540.21	0.27	-10105
5	Valence	4	2		1 (self)	-9692.06	20184.12	**21487.12**	0.27	-10290
6	Valence	5	2	1	1	-9507.37	20014.74	21643.49	0.29	-10026
7	Self/other valence	5	4			-9568.46	20136.92	21765.67	0.28	-10074.8
8	Self/other valence	6	4	1		-9336.39	19872.78	21827.28	0.30	-9953.8
9	Self/other valence	6	4	1 (self)		-9383.95	19967.91	21922.40	0.29	-9970
10	Self/other valence	6	4		1	-9316.28	19832.56	21787.06	0.30	-9952.7
11	Self/other valence	6	4		1 (self)	-9373.80	19947.61	21902.10	0.30	-9910
**12**	**Self/other valence**	**7**	**4**	**1**	**1**	**-9215.91**	**19831.82**	22112.06	**0.31**	**-9858.4**
13	Self/other asymmetric	4	3			-9799.56	20399.12	21702.11	0.26	-10032
14	Self/other asymmetric	5	3	1		-9949.23	19898.46	21527.21	0.29	-9951.2
15	Self/other asymmetric	5	3	1 (self)		-9621.75	20243.50	21872.25	0.28	-9987
16	Self/other asymmetric	5	3		1	-9460.40	19920.80	21549.54	0.29	-10017
17	Self/other asymmetric	5	3		1 (self)	-9602.57	20205.14	21833.89	0.28	-10021
18	Self/other asymmetric	6	3	1	1	-9333.66	19867.32	21821.82	0.30	-9861.5
19	Self-valence	3	2			-10058.27	20716.54	21693.79	0.24	-10120.2
20	Self-valence	4	2	1		-9774.68	20349.36	21652.36	0.27	-10098.6
21	Self-valence	4	2	1 (self)		-9904.05	20608.10	21911.10	0.26	-10187
22	Self-valence	4	2		1	-9775.09	20350.18	21653.17	0.27	-10096
23	Self-valence	4	2		1 (self)	-9908.04	20616.08	21919.08	0.26	-10032
24	Self-valence	5	2	1	1	-9637.36	20274.72	21903.47	0.28	-9966

Belief-Update Model Descriptions

Belief models contained α and β trait parameters, memory parameters η and one decision variability parameter, τ. A full list of belief-update (BU) models is given in Table S2.

Assuming that a participant used an effective number of N items of information, of which at least one was positive and one negative, then asymptotic consistency (if only positive or only negative information were presented for very many trials) meant that the update equations for the state component of beliefs took the form:

$$\alpha_{state,t+1} = (1-\eta)\alpha_{state,t} + \eta + o_t \tag{5}$$

$$\beta_{state,t+1} = (1-\eta)\beta_{state,t} + \eta + (1-o_t) \tag{6}$$

We did not use additional parameters to weigh the feedback information o_t and $1 - o_t$ to limit over-parameterising the model, allowing us to test whether preferential learning from positive and negative outcomes could simply be accounted for by activated evidence about the self, α_{trait} and β_{trait} as described in the main text. Belief-update self/other (BU S/O) models thus contained $\alpha_{self}/\beta_{self}$ and $\alpha_{other}/\beta_{other}$ trait parameters. The memory parameter was either the same for self/other, η, or separate (BU S/O full) $(\eta_{self}, \eta_{other})$.

Initial values

1. Trait belief. We can initialise each persona's starting α and β values to be the trait parameter values. This formalizes the intuition that in the absence of evidence, individuals may fall back to their default, stable beliefs. In the case of the separate self/other trait models, this meant that for the self and other personas, people will have different starting points. At trial 1, choice would only depend on the individual's trait values. By trial 2, some evidence accumulated and policy now according to ($\alpha_{state}, \beta_{state}$ + traits). At the start of each new persona, the α_{state} values reset and policy was again evaluated through just the trait values.

2. Initial bias(1). Models had separate initial starting beliefs applied to the first round of the experiment, formalizing the intuition that individuals may activate modifiable as well as trait-like components to their schemata upon entering a new environment which differ from their general traits. At trial 1 (t=1) policy was evaluated according to free parameters α_{init} and β_{init} and default state values (1).

$$\alpha_{t=1} = \alpha_{init} + \alpha_{state} \tag{7}$$

$$\beta_{t=1} = \beta_{init} + \beta_{state} \tag{8}$$

At trial 2, we assumed that individuals converged to policies determined by (accumulated evidence + traits).

3. Initial bias(2). Models again had separate initial beliefs, this time decaying over time.

$$\alpha_{t=1} = \alpha_{init} + \alpha_{trait} \tag{9}$$

$$\beta_{t=1} = \beta_{init} + \beta_{trait} \tag{10}$$

4. Fixed uncertainty. Here, we assumed a maximally uncertain starting state, given by beta distribution, $Beta(1, 1)$. Again, we applied this only to trial 1.

Belief-Update Link Functions

We explored different ways in which belief uncertainty might impact choice variability.

1. Expectation only policy. Here, we assumed that belief uncertainty has no impact on choice variability. The individual chose solely on the basis of the expectation of each option, $\alpha/(\alpha+\beta)$, $\beta/(\alpha+\beta)$ and a fixed decision noise τ, via a standard softmax function:

$$\pi_{+} = 1/(1 + \exp \frac{\beta_{eval} - \alpha_{eval}}{\tau(\beta_{eval} + \alpha_{eval})}) \tag{11}$$

2. Strength-of-evidence based policy

Here, choice became more deterministic with the amount of evidence accumulated in favour of one or the other option, i.e. as $|\alpha - \beta|$ increased:

$$\pi_{+} = 1/[1 + \exp((\beta_{eval} - \alpha_{eval})/\tau)] \quad (12)$$

3. Belief uncertainty using sampling

Here policy was randomly sampled from a distribution which was that of belief itself, albeit spread out through the decision noise τ:

$$\begin{aligned} \alpha_{\pi} &= \alpha_{eval}^{1/\tau} \\ \beta_{\pi} &= \beta_{eval}^{1/\tau} \\ \pi &\sim Beta(\alpha_{\pi}, \beta_{\pi}) \end{aligned} \quad (13)$$

The Expectation-only policy, which was agnostic to belief uncertainty, fitted the data best (Equation 11, Table S2. Belief uncertainty did not further increase decision noise. Given the absence of evidence for an effect at this level, we opted to not run this models hierarchically.

Table S2

List of belief-update models. Each also contained one decision variability parameter, τ.

n.f. refers to models not fit hierarchically.

#	Model	NP	α, β	η	α_0, β_0	Link	Initial	LL	AIC	BIC	pseudo r^2	LOO
1	BU	4	1	1		11	Trait	-9704.08	20208.16	**21511.16**	0.27	-9962
2	BU	4	1	1		11	Uncert	-9747.96	20295.93	21598.93	0.27	-9969.1
3	BU	6	1	1	1	11	IB (1)	-9630.34	20460.68	22415.18	0.28	-9954.1
4	BU	6	1	1	1	11	IB (2)	-9630.47	20460.95	22415.44	0.28	-9958.6
5	BU	4	1	1		12	Trait	-9886.91	20573.83	21876.82	0.26	n.f.
6	BU	4	1	1		12	Uncert	-9915.74	20631.48	21934.48	0.25	n.f.
7	BU	6	1	1		12	IB (1)	-9796.54	20793.08	22747.57	0.26	n.f.
8	BU	4	1	1		13	Trait	-12783.94	26367.88	27670.88	0.04	n.f.
9	BU	4	1	1		13	Uncert	-13854.33	28508.65	29811.65	-0.04	n.f.
10	BU	6	1	1	1	13	IB (1)	-13325.75	27851.50	29806.00	0.00	n.f.
11	BU S/O	6	2	1		11	Trait	-9319.84	**19839.68**	21794.17	0.30	-10486
12	BU S/O	6	2	1		11	Uncert	-9350.03	19900.05	21854.55	0.30	-10044
13	BU S/O	8	2	1	1	11	IB (1)	-9230.29	20060.58	22666.57	**0.31**	-9777.5
14	BU S/O	8	2	1	1	11	IB (2)	-9230.05	20060.11	22666.11	**0.31**	-9768.2
15	BU S/O	6	2	1		12	Trait	-9601.99	20403.98	22358.48	0.28	n.f.
16	BU S/O	6	2	1		12	Uncert	-9620.43	20440.87	22395.37	0.28	n.f.
17	BU S/O	8	2	1	1	12	IB (1)	-9501.19	20602.39	23208.39	0.29	n.f.
18	BU S/O	6	2	1		13	Trait	-13375.24	27950.49	29904.98	-0.01	n.f.
19	BU S/O	6	2	1		13	Uncert	-14540.21	30280.42	32234.92	-0.09	n.f.
20	BU S/O	8	2	1	1	13	IB (1)	-13403.15	28406.29	31012.29	-0.01	n.f.
21	BU S/O full	7	2	2		11	Trait	-9267.28	19934.57	22214.81	0.30	-10305
22	BU S/O full	7	2	2		11	Uncert	-9292.34	19984.67	22264.92	0.30	-10021.3
23	BU S/O full	9	2	2	1	11	IB (1)	-9173.31	20146.62	23078.36	**0.31**	-9776.8
**24**	**BU S/O full**	**9**	**2**	**2**	**1**	**11**	**IB (2)**	**-9173.01**	20146.03	23077.78	**0.31**	**-9761.7**
25	BU S/O full	7	2	2		12	Trait	-9456.28	20312.57	22592.81	0.29	n.f.
26	BU S/O full	7	2	2		12	Uncert	-9474.01	20348.02	22628.26	0.29	n.f.
27	BU S/O full	9	2	2	1	12	IB (1)	-9357.03	20514.06	23445.80	0.30	n.f.
28	BU S/O full	7	2	2		13	Trait	-12979.24	27358.47	29638.72	0.02	n.f.

Self-schema: from intuition to key computational hypothesis

Inspired by the work of Koban et al. (2017), but also by the cognitive-behavioural hypothesis that activated, schema-based beliefs about the self appear to 'filter' information and facilitate congruent learning, we performed simulation experiments to establish *prima facie* validity of hypotheses. We set up a model based on the schema-based formulation, and asked whether such a model would behave, from an associative learning point of view, as if it learnt faster about congruent information. Here, we demonstrate the key simulation that led to the hypothesis tested in the main analysis.

In this demonstration, a person accumulates evidence about how two raters like them, one 80% positive and one 80% negative. They simply add 1 to their positive tally, α if they perceive a positive observation from social feedback $o_t = 1$, or 1 to their negative tally β, if they receive disapproving feedback $o_t = 0$.

$$\begin{aligned} \alpha_t &\leftarrow \alpha_{t-1} + o_t \\ \beta_t &\leftarrow \beta_{t-1} + 1 - o_t \end{aligned} \tag{14}$$

However our person has an amount of 'active background evidence' about themselves. For example, they already have positive information weighing as much as 10 items of positive observations in mind, and 2 items of negative information active about themselves. This is our proxy for the positive self bias in the active schema; however it could be reinterpreted as an assumption about whatever the object of learning is, depending on the experiment. So their *total* active evidence is:

$$\begin{aligned} \alpha_{tot} &\leftarrow \alpha_0 + \alpha_{t-1} + o_t \\ \beta_{tot} &\leftarrow \beta_0 + \beta_{t-1} + (1 - o_t) \end{aligned} \tag{15}$$

Clinically, people seem to have self-schemas persistent in their influence and hard to overcome by new evidence, maybe because new information is often easily forgotten. We wondered if corresponds to Rescorla-Wagner learning, where the learning rate inherently induces *replacement*, that is, forgetting, of old association values with

prediction-error based ones. We translated this clinical and computational intuition to the self-evaluation setting by assuming that new evidence, whether of approval or disapproval, is imperfectly remembered from trial to trial with a memory parameter $0 < m < 1$, giving:

$$\begin{aligned} \alpha_{tot} &\leftarrow \alpha_0 + m\,\alpha_{t-1} + o_t \\ \beta_{tot} &\leftarrow \beta_0 + m\,\beta_{t-1} + (1 - o_t) \end{aligned} \quad (16)$$

For the purposes of demonstration, we write the memory parameter in its simplest form and provide further explanation for the form actually used in the main work in equations 21 - 23 below. Finally, CBT-like belief strength in this simple model is simply the proportion of active evidence about the statement in question:

$$\begin{aligned} \mu_+ &= \alpha_{tot}/(\alpha_{tot} + \beta_{tot}) \\ \mu_- &= \beta_{tot}/(\alpha_{tot} + \beta_{tot}) \end{aligned} \quad (17)$$

Having formulated this simple model of evidence accumulation into beliefs, how can we map it onto a value-based, associative model? We could complete the simulation by linking beliefs to behaviour, and fitting this behaviour with an associative model, but greater conceptual clarity can be achieved as follows. In our task, we take the choice probabilities π to scale according to belief strength in a schema-model, but with $exp(action\ value)$ in a value-based model (consistent with, for example, the standard Gibbs Softmax):

$$\begin{aligned} \mu &\sim \pi \sim e^Q \text{ so, for example,} \\ Q_+ &= \ln(\mu_+) = \ln(\alpha_{tot}/(\alpha_{tot} + \beta_{tot})) \end{aligned} \quad (18)$$

(We have omitted proportionality constants which do not materially affect this demonstration). Next, we can solve the Rescorla-Wagner learning rule as if the learning rate were the unknown, and obtain for each belief update in the cognitive model the

equivalent associative learning rate:

$$\begin{aligned} Q_{t+1} &= Q_t + \lambda(r_t - Q_t) \Rightarrow \\ \lambda &= \frac{Q_{t+1} - Q_t}{r_t - Q_t} \\ &= \frac{\ln \mu_{t+1} - \ln \mu_t}{r_t - \ln \mu_t} \end{aligned} \tag{19}$$

These effective learning rates can be averaged to demonstrate if indeed they are higher for in the presence of $\alpha_0 > \beta_0$. This lead to the hypothesis that in the real data higher learning rates would indeed correspond to congruent activated schemas, with more positive or less negative information activated about the self and either of the two being relatively blunted in those with high FNE.

Note, however, that the in this demonstration only self-schema parameters are considered, whereas in principle models fitted to the real data contained several more parameters that might account for descriptive differences in behaviour - or even fail to capture these altogether. Note also that the 'equivalent associative learning rate' above does not map exactly to the λ_{pos} and λ_{neg} in our associative models above.

Further details on belief models

Memory, or the leaky accumulation of evidence

Participants may gradually forget older evidence, as well as accumulate new evidence, about themselves. We thus took the evidence α_t and β_t to be subject to decay, as well as trial-dependent accumulation.

Furthermore, we wanted α_t and β_t to be no lesser than 1. If this condition is met, the belief distribution has a value of 0 at the extremes $p = 0$ and $p = 1$. This formalizes the commonsense assumption that people do not think that their most likely self-evaluation is perfectly positive, nor perfectly negative. U-shaped beliefs are also possible in principle, but here we adopted a more conservative framework.

We now consider how participants may update information in their working memory by partially forgetting older information. We denote the maximum size of effective memory to be $N = max(\alpha_t + \beta_t)$. We also need the beliefs represented by α_t

and β_t to make sense if participants do not - as yet, or through memory decay - have any evidence about the current context. We assume that they then become agnostic about how they will be evaluated by others, which is achieved by $\alpha_t = \beta_t = 1$. If now $\alpha_t, \beta_t \geq 1$. Happily, this also excludes nonsensical values for α_{total} and β_{total}. If n^+, n^- is the positive and negative evidence retained from the task itself, over and above the minimum of 1,

$$\begin{aligned} \alpha_t &= 1 + n^+ \\ \beta_t &= 1 + n^- \end{aligned} \tag{20}$$

Consistent with traditional views of working memory update, we take old observations to be chosen at random to replace. This is on average equivalent to a forgetting process operating on α_{t-1}. We now show that this forgetting rate is fully determined by the requirement for α to tend to N-1 (so β is still at least 1) if all $o = 1$, and to 1 if all $o = 0$. Now $max(\alpha_t) = N - 1$. Only the n^+ part of α decays before updating, so for example in the absence of positive feedback $n_t^+ = (1 - \eta)n_{t-1}^+$. If the participant receives positive evidence all the time,

$$\begin{aligned} max(\alpha_t) &= [max(n_t^+)(1 - \eta) + 1] + max(o_t) \Rightarrow \\ max(\alpha_t) &= [(max(\alpha_t) - 1)(1 - \eta) + 1] + 1 \Rightarrow \\ N - 1 &= [(N - 1 - 1)(1 - \eta) + 1] + 1 \Rightarrow \\ (1 - \eta) &= (N - 3)/(N - 2) \Rightarrow \eta = 1/(N - 2) \end{aligned} \tag{21}$$

Substituting this into the update equation for α_t gives:

$$\begin{aligned} \alpha_{t+1} &= [n_t^+(1 - \eta) + 1] + o_t \Rightarrow \\ \alpha_{t+1} &= (\alpha_t - 1)(1 - \eta) + 1 + o_t \Rightarrow \\ \alpha_{t+1} &= \alpha_t(1 - \eta) + \eta + o_t \Rightarrow \\ &= \alpha_t \frac{N - 3}{N - 2} + \frac{1}{N - 2} + o_t \end{aligned} \tag{22}$$

When it comes to real data, η, is a free parameter to be fitted. Apart from $0 \leq \eta < 1$ (or equivalently $2 < N < \infty$) the value of η is not constrained (though it can be subject to regularizing or empirical priors). It is directly related to the effective memory. β_{t+1} is estimated in the exact same manner:

$$\beta_{t+1} = (1 - \eta)\beta_t + \eta + (1 - o_t) \tag{23}$$

Finally, we note that in the models fitted here o_t is either 0 or 1. In future work, we agents can be considered that over-count or under-count information.

Parameter recovery

Correlations between fitted parameter values for self/other asymmetric model the self/other belief-update model and recovered parameter values. Parameter recovery was performed by taking the best-fitting parameters as derived by the MCMC hierarchical fits with the key parameter FNE correlations embedded and bootstrapping 1000 values, generating synthetic data and then re-fitting it. Initial values for both models were set to the mean parameter values derived from the MCMC fits and informed by the bounds of each parameter.

Table S3

Parameter recovery correlations taken from a bootstrapped sample of 1000 parameter values. Key parameters from each model shown in bold.

Associative Learning Parameter	Spearman's Rho, p value	Belief-update Parameter	Spearman's Rho, p value
Self-positive learning rate	r = 0.38, p <.001	**Alpha-self**	**r = 0.72, p <.001**
**Self-negative learning rate**	**r = 0.64, p <.001**	Beta-self	r = 0.29, p = < .001
Other learning rate	r = 0.72, p <.001	Alpha-other	r = 0.38, p < .001
Tau	r = 0.75, p <.001	Beta-other	r = 0.37, p <.001
Initial Bias	r = 0.54, p <.001	Memory	r = 0.74, p <.001
Positivity Bias	r = 0.22, p <.001	Tau	r = 0.78, p <.001
		Initial bias alpha	r = 0.22, p = <.001
		Initial bias beta	r = 0.21, p = <.001

Simulated key parameter differences

In order to illustrate the effects of the key parameter, $\lambda_{selfneg}$, for the best-fitting associative learning model, we input different values of the parameter and generated synthetic data for 100 participants. The rest of the parameters were set to the best-fitting parameter values as obtained by MCMC. The results are displayed in Figure S1. The same procedure was done with the α_{self} parameter from the best-fitting belief-update model, displayed in Figure S2.

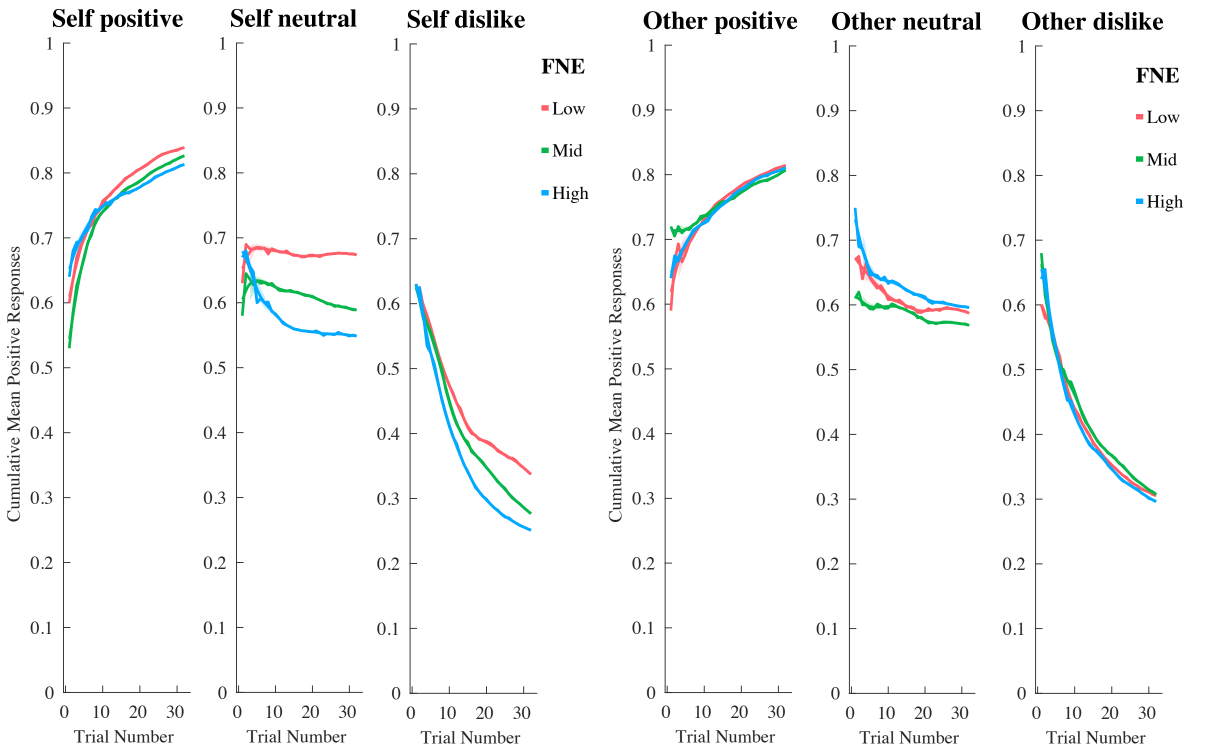

(a) (b)

Figure S1. Simulated data for different parameter values of the $\lambda_{selfneg}$ parameter from the self/other valence model. Parameter values differentiate behaviour according to FNE in self (a) conditions, but not in other (b) conditions.

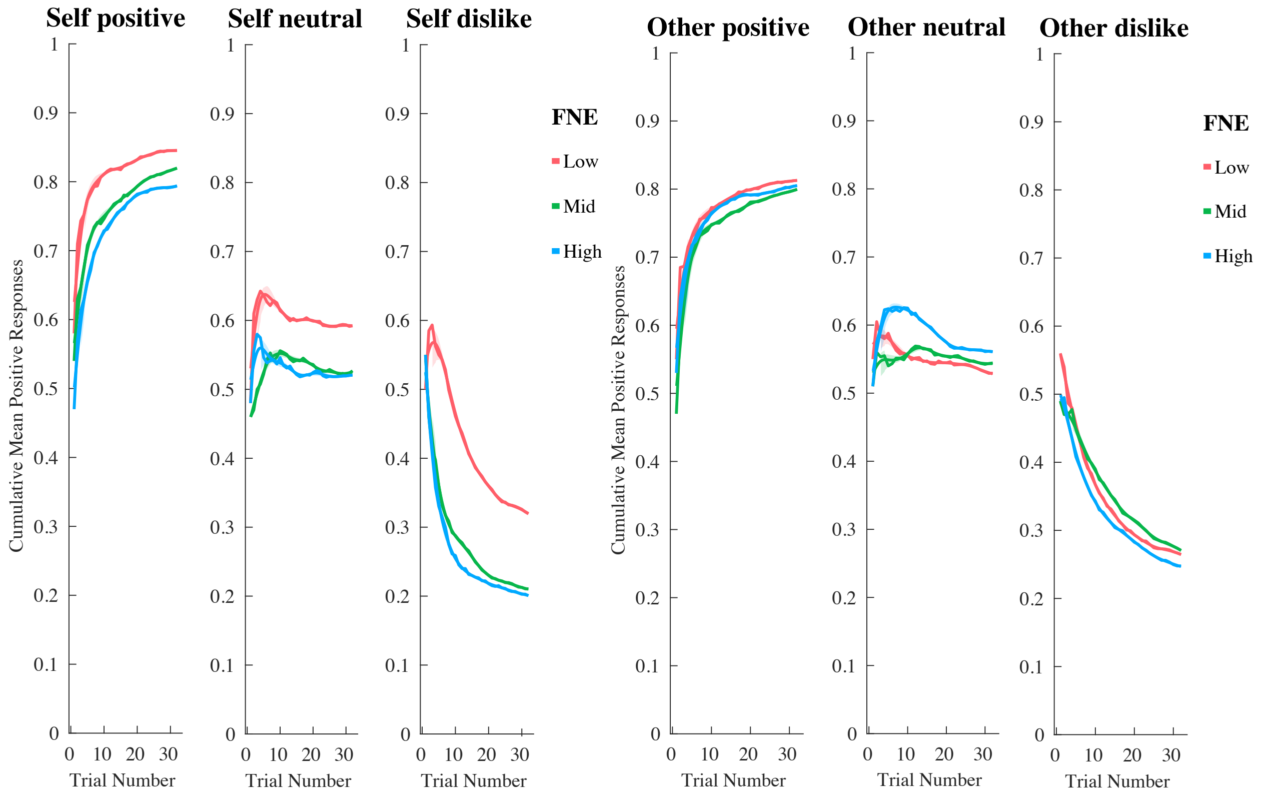

(a) (b)

Figure S2. Simulated data for different parameter values of the α_{self} parameter from the self/other belief-update model. Parameter values differentiate behaviour according to FNE in self (a) conditions, but not in other (b) conditions

Correspondence between associative learning and belief-update models

In order to determine the stability of the $\lambda_{selfneg}$, α_{self} correlation across models, we computed the correlation for each nested model variant within the best-fitting model family. Figure S3 displays the correlations between the self-negative learning rate parameter from variants of the self/other valence models and the α_{self} parameter from the best-fitting self/other belief-update models. All parameter correlations were significant and have similar values across model variants, with all displaying negative Spearman's correlations ranging from $r = -0.48$ to $r = -0.57$. Figure S4 displays the converse, thus shows variants of α_{self} across different self/other belief-update model variants correlated with the best-fitting self/other valence model parameter. Again, all parameter correlations were significant and have similar values across model variants,

with all displaying negative Spearman's correlations ranging from $r = -0.49$ to $r = -0.57$.

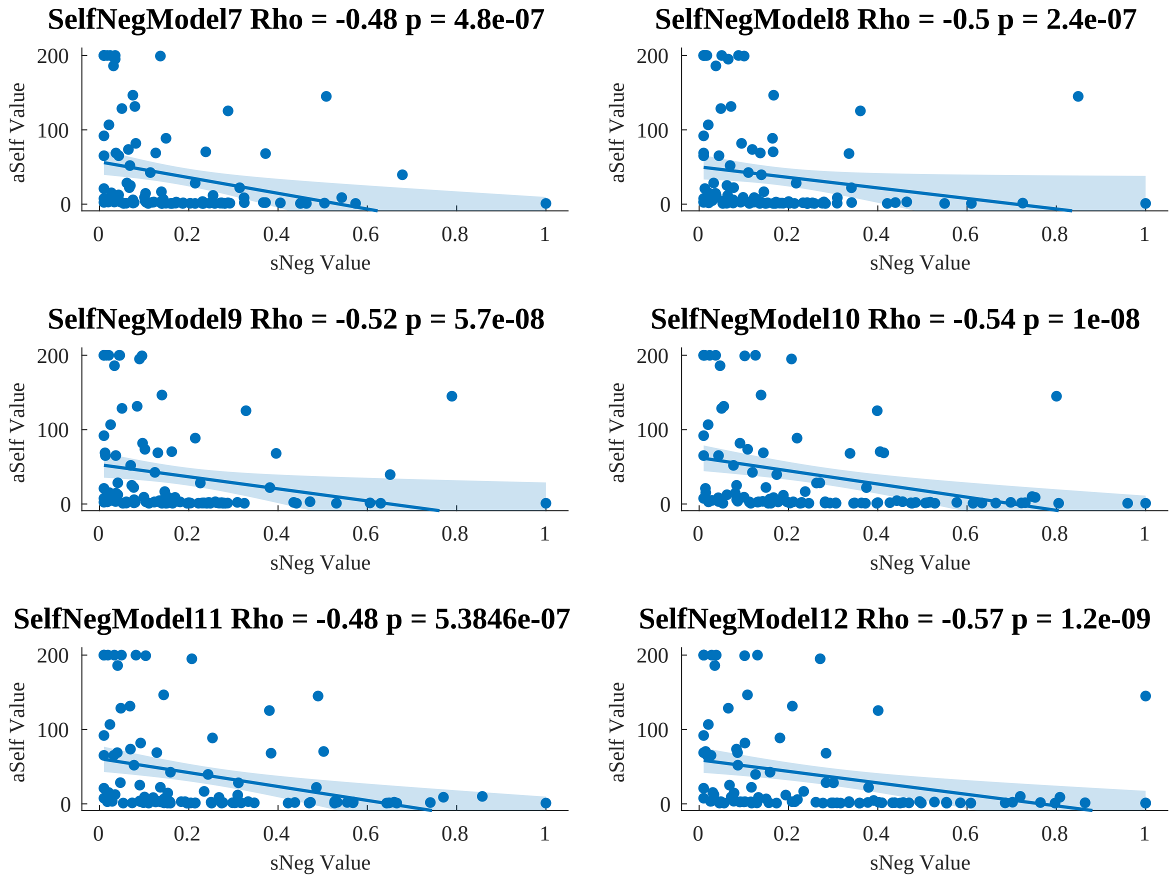

Figure S3. Correlations between the $\lambda_{selfneg}$ learning rate parameters from variants of the self/other valence models and the α_{self} parameter from the best-fitting belief-update model.

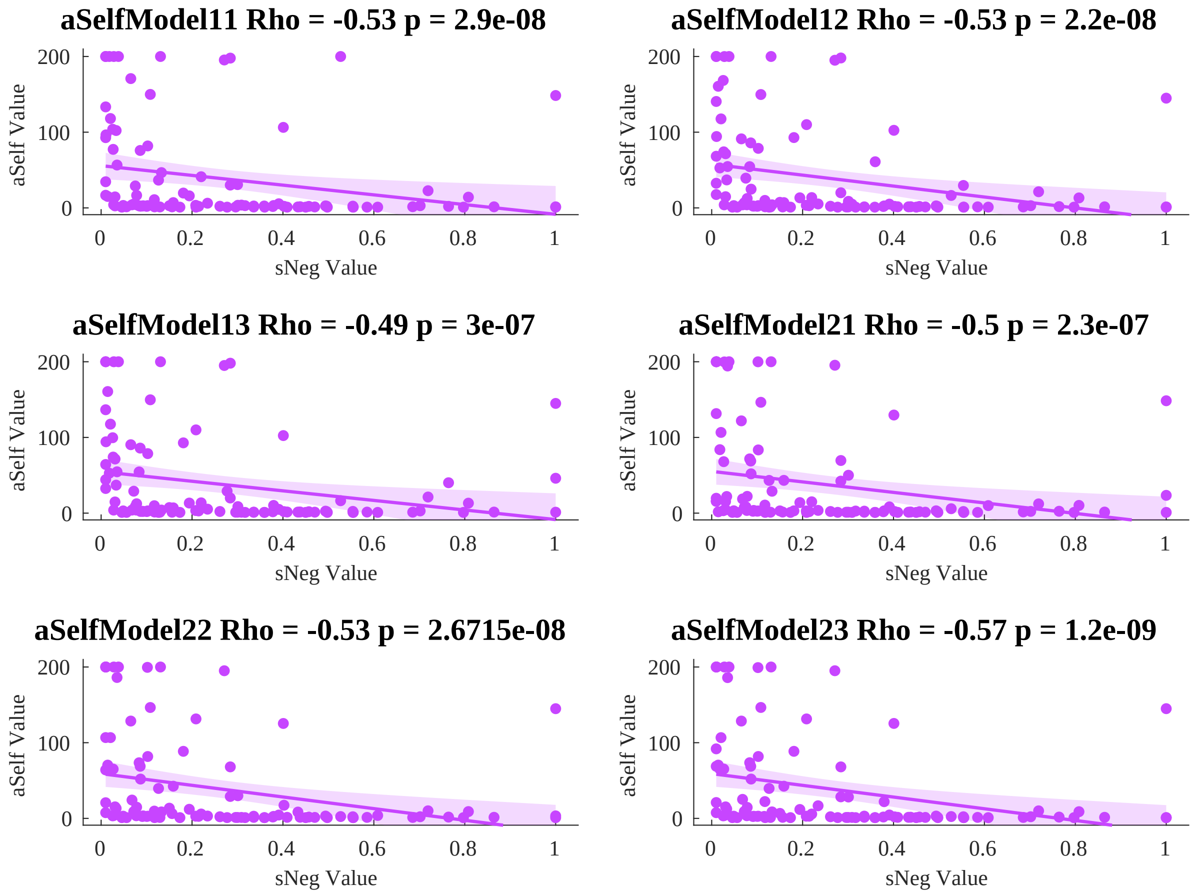

Figure S4. Correlations between the α_{self} parameters from variants of the self-other belief-update models and the $\lambda_{selfneg}$ learning rate parameter from the best-fitting self/other valence models

Generative performance of best-fitting model from other model families

Simple models

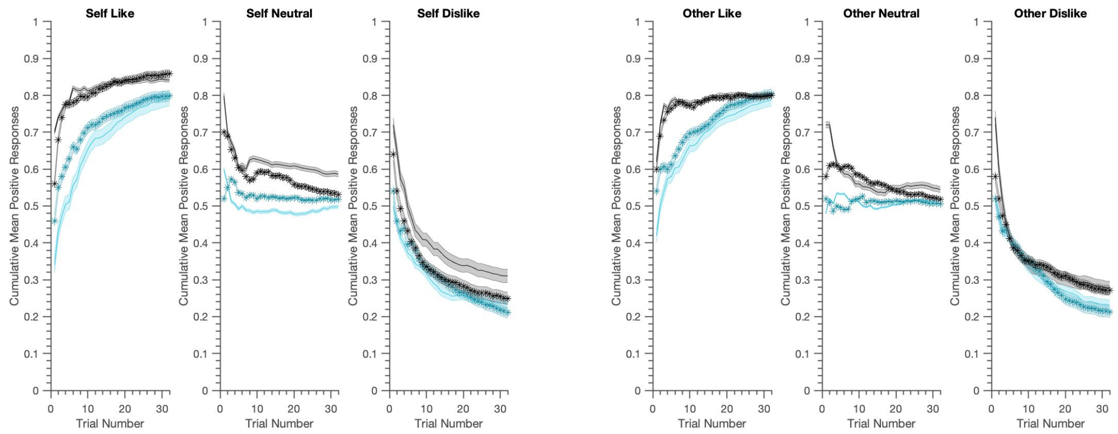

(a) (b)

Figure S5. Generative performance for the Associative Learning Valence Model (Model Number 5 Table S1); mean cumulative positive words chosen for actual data (in black) vs. data generated from MCMC fits (cyan). Data is visualised using median-split FNE scores (lighter=lower BFNE) and shaded zones represent +/− SEM. The generated data captures the asymmetries in positive vs. negative word selection but not the key high VS low FNE differences.

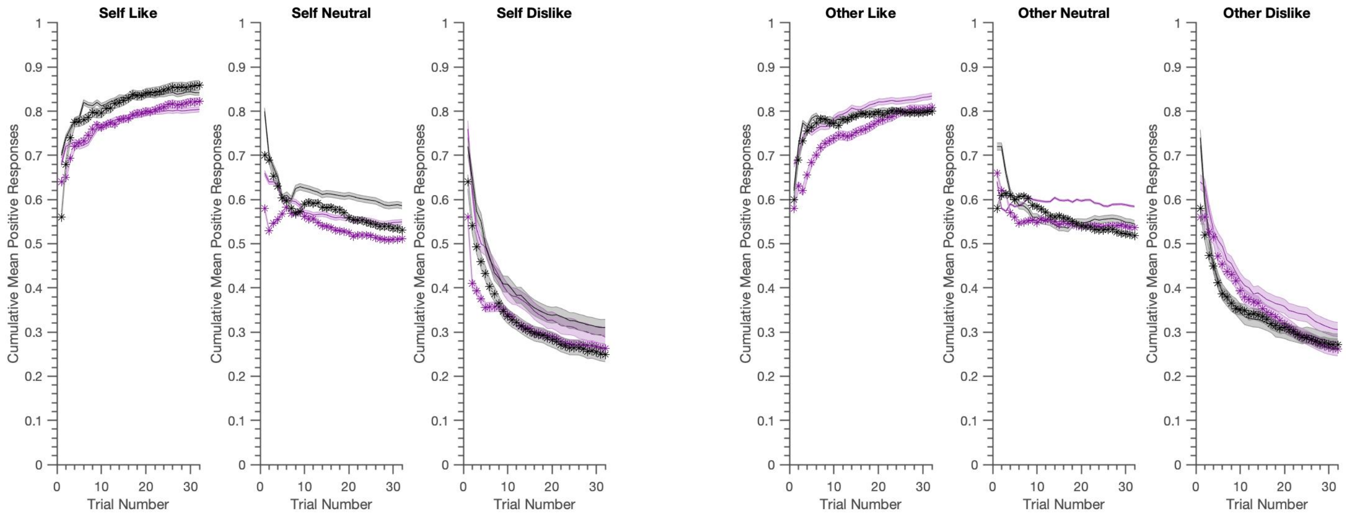

(a) (b)

Figure S6. Generative performance for the Belief-Update Simple Model (Model Number 1 Table S2); mean cumulative positive words chosen for actual data (in black) vs. data generated from MCMC fits (cyan). Data is visualised using median-split FNE scores (lighter=lower BFNE) and shaded zones represent +/− SEM. The generated data captures the asymmetries in positive vs. negative word selection but not the key high VS low FNE differences for the self/other distinction.

Table S4

Generative statistics for simple models. Both simple models display poor ability to reproduce many of the key statistics, including the 3-way-interaction.

Contrast	AL Mean β [CI]	% of sig samples	BU Mean β [CI]	% of sig samples
Main effect BFNE	-0.08 [-0.07 -0.09]	10	-0.27 [-0.26 -0.28]	98
Main effect self/other	-0.04 [-0.28 0.20]	0	-0.21 [-0.52 0.10]	3
Main effect persona: like	50.67 [50.11 51.23]	100	28.3 [27.55 29.04]	100
Main effect persona: neutral	25.37 [24.55 26.20]	100	15.99 [15.23 16.74]	98
BFNE X self/other	0.01 [-0.82 0.83]	0	0.01 [-0.01 0.01]	2
BFNE X persona: like	0.17 [0.16 0.18]	30	0.31 [0.29 0.32]	87
BFNE X persona: neutral	0.08 [0.06 0.10]	9	0.13 [0.11 0.15]	22
BFNE X self/other X persona	-0.01 [-0.02 0.02]	1	-0.01 [-0.02 0.01]	1

Full models

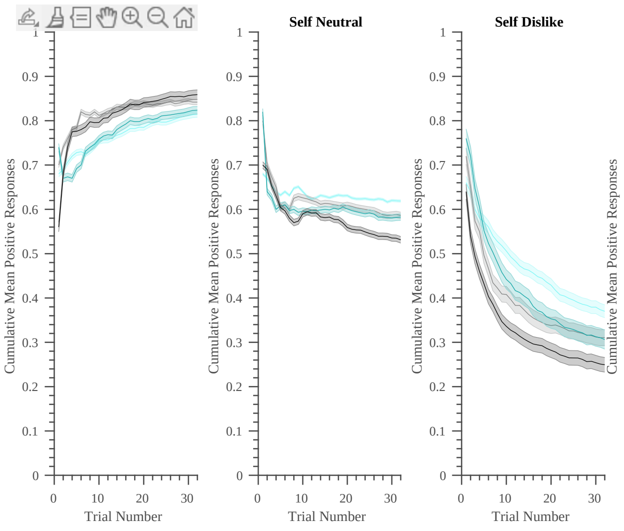

(a)

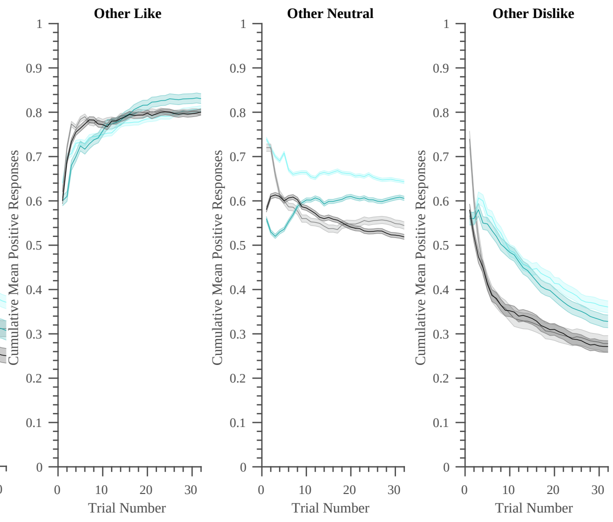

(b)

Figure S7. Generative performance for the Associative Learning S/O Valence Model (Model Number 12 Table S1); mean cumulative positive words chosen for actual data (in black) vs. data generated from MCMC fits (cyan). Data is visualised using median-split FNE scores (lighter=lower BFNE) and shaded zones represent +/− SEM. The generated data captures the asymmetries in positive vs. negative word selection and the key high VS low FNE differences, similarly to Model 18

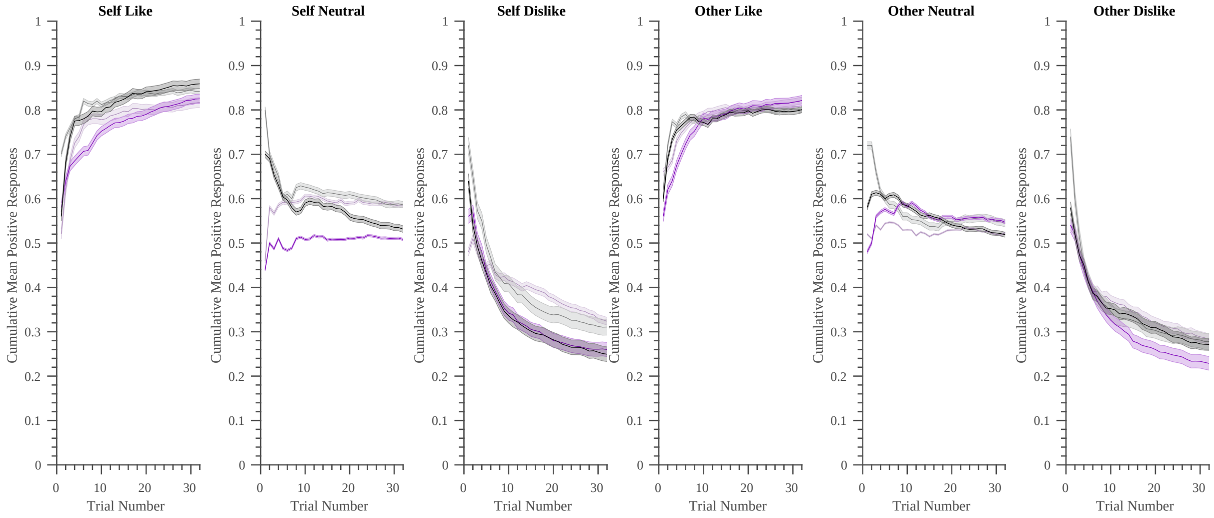

(a) (b)

Figure S8. Generative performance for the Belief-update self-other full model (Model Number 24 Table S2); mean cumulative positive words chosen for actual data (in black) vs. data generated from MCMC fits (mauve). Data is visualised using median-split FNE scores (lighter=lower BFNE) and shaded zones represent +/− SEM. The generated data captures the asymmetries in positive vs. negative word selection and the key high VS low FNE differences, similarly to Model 14

Table S5

Generative performance statistics from full models.

	Associative learning model		Belief-update model	
Contrast	Mean β coefficient	% of sig samples	Mean β coefficient	% of sig samples
Main effect BFNE	-0.66 [-0.67 -0.65]	100	-0.73 [-0.74 -0.72]	100
Main effect self/other	-10.76 [-11.04 -10.49]	100	-13.45 [-13.76 -13.14]	100
Main effect persona: like	21.68 [21.09 22.27]	100	25.61 [25.08 26.13]	100
Main effect persona: neutral	16.86 [16.16 17.56]	98	15.61 [14.80 16.41]	92
BFNE X self/other	0.27 [0.26 0.28]	100	0.27 [0.27 0.28]	100
BFNE X persona: like	0.63 [0.62 0.64]	100	0.72 [0.71 0.73]	100
BFNE X persona: neutral	0.16 [0.14 0.17]	22	0.28 [0.26 0.30]	64
BFNE X self/other X persona	-0.22 [-0.22 -0.23]	98	-0.23 [-0.24 0.22]	94

[a]Note: [Lower CI Upper CI 95%]

Table S6

Parameter weights on FNE from the full models, derived from clinically informed model-fitting. The key FNE parameter differences were the same as for the selected models.

Associative Learning parameter	Mean w [Lower CI − Upper CI 95%]	Belief-update parameter	Mean w [Lower CI − Upper CI 95%]
$\lambda_{self,+ve}$	0.01 [−0.09 0.10]	α_{self}	**−0.44 [−0.80 −0.04]**
$\lambda_{self,-ve}$	**0.11 [0.01 0.22]**	β_{self}	−0.04 [−0.73 0.81]
$\lambda_{other,+ve}$	−0.06 [−0.14 0.03]	α_{other}	−0.05 [−0.18 0.09]
$\lambda_{other,-ve}$	−0.01 [−0.08 0.07]	β_{other}	0.14 [−0.24 0.52]
τ	−0.01 [−0.08 0.07]	η_{self}	−0.33 [−0.79 0.13]
Initial bias	−0.58 [−1.22 0.07]	η_{other}	−0.10 [−0.47 0.26]
Pos. bias	−0.04 [−0.19 0.10]	τ	−0.09 [−0.25 0.06]
		$\alpha_{initial}$	−0.14 [−0.32 0.04]
		$\beta_{initial}$	−0.09 [−0.04 0.22]

R demo code

Link to github repository: `https://github.com/alexxxkh/SocialEvaluation`.

Demonstration code in R corresponding to section on developing the Self-schema hypothesis.

```
totTrN = 30;  # Our 'task' will consist of this many trials with an 80% positive rater and
              # as many again with an 80% negative rater.
totPtN = 200; # 200 simulated participants (with same parameters)
# Belief parameters :
ptBelPar = array(NA,c(totPtN,3));
colnames(ptBelPar)=c('alph0','bet0','mem');

# All pts have same params, for convenience:
ptBelPar[,'alph0'] = 10; ptBelPar[,'bet0'] = 2;          # 'ballast' sizes.
ptBelPar[,'mem'] = 10;   # effecive size of buffer available for inferring



RV = array(NA,c(totTrN,10,totPtN));
colnames(RV) = c('rpos','vposhi','vposlo','dvpos','rneg',
                'vneghi','vneglo','dvneg','lrpos','lrneg');
efeta = list();
lrnR = array(NA,c(totPtN,2)); colnames(lrnR) = c('bte','wte');

for (ptN in 1:totPtN) {
   efeta[[ptN]] = list();
   RV[,'rpos',ptN] = sample(c(0,1),totTrN,replace=TRUE,prob=c(0.2,0.8));
   RV[,'rneg',ptN] = sample(c(0,1),totTrN,replace=TRUE,prob=c(0.8,0.2));
   m = 1 - 1/ptBelPar[ptN,'mem'];   # remembering rate corresponding to
                                    # effective working memory size
```

```
# Baseline beliefs / values :
a0 = ptBelPar[ptN,'alph0'] ; b0 = ptBelPar[ptN,'bet0'];
v0hi = log(a0 / (a0 + b0));
v0lo = log(b0 / (a0 + b0));
dv0 = v0hi - v0lo;


a = c(0,0); b=c(0,0)

for (trN in 1:totTrN) {
  # trials with positive rater
  a[1] = m*a[1] +     RV[trN,'rpos',ptN];    # acccum. +ve returns by positive rater
  b[1] = m*b[1] + 1 - RV[trN,'rpos',ptN];    # acccum. -ve returns by positive rater
  RV[trN,'vposhi',ptN] = log((a0 + a[1])/ (a0 + a[1] + b0 + b[1]));
  RV[trN,'vposlo',ptN] = log((b0 + b[1])/ (a0 + a[1] + b0 + b[1]));
  RV[trN,'dvpos',ptN] = log((a0 + a[1])/ (b0 + b[1]));

  # Now to calculate the lr that an RW agent would have to end up with
  # the same value update. NB 'pos' and 'neg' refer to the positive and negative
  # raters, NOT better-than-expected vs worse-than-expected prediction errors.
  if (RV[trN,'rpos',ptN] > 0.5)  {  # ##########  i.e. BTE trial ################
    if (trN == 1){    # for first trial only
      RV[trN,'lrpos',ptN] =  (RV[trN,'dvpos',ptN] - dv0) / (1- RV[trN,'vposhi',ptN]) ;
    } else {          # for most trials
      RV[trN,'lrpos',ptN] =  (RV[trN,'dvpos',ptN] - RV[trN-1,'dvpos',ptN]) /
                             (1- RV[trN,'vposhi',ptN]) ;
    }
  }    else   {   # ########################   WTE trial ########################
    if (trN == 1){    # for first trial only
```

```
    RV[trN,'lrpos',ptN] =  -(RV[trN,'dvpos',ptN] - dv0) / (1- RV[trN,'vposlo',ptN]) ;
  } else {          # for most trials
    RV[trN,'lrpos',ptN] =  -(RV[trN,'dvpos',ptN] - RV[trN-1,'dvpos',ptN]) /
                            (1- RV[trN,'vposlo',ptN]) ;
  }

}

# trials with negative rater
a[2] = m*a[2] +     RV[trN,'rneg',ptN];    # acccum. +ve returns by negative rater
b[2] = m*b[2] + 1 - RV[trN,'rneg',ptN];    # acccum. -ve returns by negative rater
RV[trN,'vneghi',ptN] = log((a0 + a[2])/ (a0 + a[2] + b0 + b[2]));
RV[trN,'vneglo',ptN] = log((b0 + b[2])/ (a0 + a[2] + b0 + b[2]));
RV[trN,'dvneg',ptN] = log((a0 + a[2])/ (b0 + b[2]));
# Again, calculate the lr that an RW agent would have to end up with the
# same value update.
if (RV[trN,'rneg',ptN] > 0.5)  {  # ##########  i.e. BTE trial ################
  if (trN == 1){    # for first trial only
    RV[trN,'lrneg',ptN] =  (RV[trN,'dvneg',ptN] - dv0) /
                           (1- RV[trN,'vneghi',ptN]) ;
  } else {          # for most trials
    RV[trN,'lrneg',ptN] =  (RV[trN,'dvneg',ptN] - RV[trN-1,'dvneg',ptN]) /
                           (1- RV[trN,'vneghi',ptN]) ;
  }
}    else   {   # ########################   WTE trial ########################
  if (trN == 1){    # for first trial only
    RV[trN,'lrneg',ptN] =  -(RV[trN,'dvneg',ptN] - dv0) /
                            (1- RV[trN,'vneglo',ptN]) ;
  } else {          # for most trials
```

```
        RV[trN,'lrneg',ptN] =  -(RV[trN,'dvneg',ptN] - RV[trN-1,'dvneg',ptN]) /
                                (1- RV[trN,'vneglo',ptN]) ;
      }
    }
  }

  efeta[[ptN]][[1]] <- c( RV[ RV[,'rpos',ptN] > 0.5,'lrpos',ptN] ,
                          RV[ RV[,'rneg',ptN] > 0.5,'lrneg',ptN]  );
  efeta[[ptN]][[2]] <- c( RV[ RV[,'rpos',ptN] < 0.5,'lrpos',ptN] ,
                          RV[ RV[,'rneg',ptN] < 0.5,'lrneg',ptN]  );
  names(efeta[[ptN]]) <- c('bte','wte');
  lrnR[ptN,] <- c( mean(na.omit(efeta[[ptN]][[1]])),  mean(na.omit(efeta[[ptN]][[2]])) ) ;

}

plot(RV[,'vposhi',1],ylim=c(-1,0),t='l',col='blue',xlab='trial',
     main='blue: Q(pos. word), "like" rater \n red: Q(pos. word), "dislike" rater')
lines(RV[,'vneghi',1],ylim=c(-1,0),t='l',col='red',xlab='trial')

print('Over all simulated pts:',quote=F)
hist(lrnR[,'bte']-lrnR[,'wte'],col='grey75'); abline(v=0,col='black',lwd=3)
print(wilcox.test(lrnR[,'bte']-lrnR[,'wte']))



print('Click back for example plot',quote=F)
```

References

Akaike, H. (1998). Information Theory and an Extension of the Maximum Likelihood Principle. In E. Parzen, K. Tanabe, & G. Kitagawa (Eds.), *Selected Papers of Hirotugu Akaike* (pp. 199–213). New York, NY: Springer New York. Retrieved from https://doi.org/10.1007/978-1-4612-1694-0_15 doi: 10.1007/978-1-4612-1694-0_15

Camerer, C., & Ho, T. H. (1999). Experience-weighted Attraction Learning in Normal Form Games. *Econometrica*, *67*(4), 827–874. Retrieved 2020-08-31, from https://onlinelibrary.wiley.com/doi/abs/10.1111/1468-0262.00054 (_eprint: https://onlinelibrary.wiley.com/doi/pdf/10.1111/1468-0262.00054) doi: 10.1111/1468-0262.00054

Daw, N. D., O'Doherty, J. P., Dayan, P., Seymour, B., & Dolan, R. J. (2006, June). Cortical substrates for exploratory decisions in humans. *Nature*, *441*(7095), 876–879. Retrieved 2017-07-13, from http://www.nature.com/nature/journal/v441/n7095/full/nature04766.html doi: 10.1038/nature04766

Koban, L., Schneider, R., Ashar, Y. K., Andrews-Hanna, J. R., Landy, L., Moscovitch, D. A., … Arch, J. J. (2017, December). Social anxiety is characterized by biased learning about performance and the self. *Emotion (Washington, D.C.)*, *17*(8), 1144–1155. Retrieved 2019-05-17, from https://www.ncbi.nlm.nih.gov/pmc/articles/PMC5623172/ doi: 10.1037/emo0000296

Schwarz, G. (1978). Estimating the Dimension of a Model. *The Annals of Statistics*, *6*(2), 461–464. Retrieved 2019-07-23, from https://www.jstor.org/stable/2958889

Vehtari, A., Gelman, A., & Gabry, J. (2017, September). Practical Bayesian model evaluation using leave-one-out cross-validation and WAIC. *Statistics and Computing*, *27*(5), 1413–1432. Retrieved 2020-08-30, from https://doi.org/10.1007/s11222-016-9696-4 doi: 10.1007/s11222-016-9696-4
